# Design, synthesis, and antiproliferative activity of novel thiazole-based derivatives as tubulin polymerization inhibitors targeting the colchicine binding site

**DOI:** 10.1039/d6ra01355d

**Published:** 2026-05-12

**Authors:** Lamya H. Al-Wahaibi, Ali M. Elshamsy, Taha F. S. Ali, Bahaa G. M. Youssif, Stefan Bräse, Mohamed Abdel-Aziz, Nawal A. El-Koussi

**Affiliations:** a Department of Chemistry, College of Sciences, Princess Nourah Bint Abdulrahman University Riyadh 11671 Saudi Arabia; b Pharmceutical Chemistry Department, Faculty of Pharmacy, Deraya University Minia Egypt; c Medicinal Chemistry Department, Faculty of Pharmacy, Minia University Minia 61519 Egypt abulnil@hotmail.com +2101003311327; d Department of Pharmaceutical Organic Chemistry, Faculty of Pharmacy, Assiut University Assiut-71526 Egypt bgyoussif2@gmail.com +201098294419; e Institute of Biological and Chemical Systems, IBCS-FMS, Karlsruhe Institute of Technology 76131 Karlsruhe Germany braese@kit.edu; f Medicinal Chemistry Department, Faculty of Pharmacy, International Minia University Minia Egypt; g Department of Pharmaceutical Medicinal Chemistry, Faculty of Pharmacy, Assiut University Assiut Egypt

## Abstract

The development of novel microtubule-targeting medicines (MTAs) remains a crucial strategy in cancer treatment, as they combat drug resistance and systemic toxicity. A novel series of thiazole-based derivatives was synthesized, characterized, and evaluated as antitubulin agents endowed with antiproliferative action. An IC_50_ experiment was performed to assess the efficacy of novel compounds 9a–o in suppressing tubulin activity. The antiproliferative effects of the most potent compounds were evaluated. The levels of initiator caspases (Caspase-8 and Caspase-9) and executioner caspase (Caspase-3) were examined to ascertain the degree of apoptosis. Additionally, the expression levels of the mitochondrial regulatory proteins Bax and Bcl-2 were examined to ascertain the importance of the intrinsic apoptotic pathway. Compound 9k exhibited significant inhibition of tubulin with an IC_50_ of 1.56 µM and showed potent activity against HeLa (cervical), HCT-116 (colorectal), and A-549 (lung) cancer cell lines. The apoptotic assays revealed that 9k effectively triggered the apoptotic cascade, leading to a ninefold increase in Caspase-3 and a significant twenty-onefold rise in Caspase-9, surpassing the effects of the reference Staurosporine. Caspase-8 was activated by an elevenfold increase, primarily *via* the intrinsic pathway. This was confirmed by a substantial change in the mitochondrial “rheostat”: 9k induced a 38-fold increase in pro-apoptotic Bax and a 5-fold decrease in anti-apoptotic Bcl-2. Molecular docking studies showed that 9k exhibited a favorable binding mode consistent with its tubulin-inhibition profile. *In silico* ADMET predictions further supported 9k as a drug-like lead with acceptable oral exposure and a beneficial P-gp–related transporter profile. The enhanced apoptotic effects of 9k compared with Staurosporine make 9k an attractive lead candidate for further development as an anti-cancer drug targeting the colchicine-binding site.

## Abstract 2

Compound 9k exhibited high potency as an antitubulin agent, with an IC_50_ value of 1.56 µM, demonstrating double the efficacy of the reference compound CA-4.

## Graphical abstract

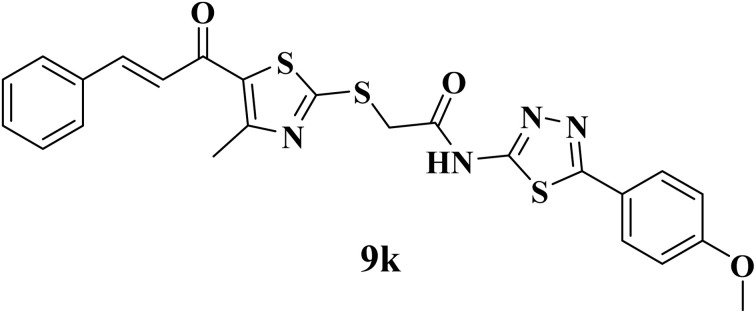

## 1. Introduction

Cancer is a huge concern in global healthcare. It occurs when cells proliferate uncontrollably and fail to undergo programmed cell death (apoptosis).^1^ A key approach to combating cancer is to modify microtubule function. This remains an essential component of chemotherapy.^2,3^ Microtubules, composed of α- and β-tubulin heterodimers, are essential for fundamental cellular processes such as intracellular transport, structural integrity, and the formation of the mitotic spindle during cell division.^4,5^

Taxanes and vinca alkaloids are tubulin inhibitors that cause cell death by controlling the assembly of microtubules. This interference triggers the spindle assembly checkpoint, which halts the cell cycle at the G2/M phase.^6,7^ However, the therapeutic efficacy of existing tubulin-targeting medicines is often limited by the development of multidrug resistance and significant systemic toxicity.^8,9^ Consequently, there is a critical need to develop new small-molecule tubulin inhibitors with strong antiproliferative effects and a distinct apoptotic mechanism.

Taxanes are therapeutically effective microtubule-targeting agents that act on polymerized microtubules.^10^ The colchicine binding site (CBS) has distinct pharmacological advantages. The CBS serves as the primary target for many small-molecule destabilizers. It is situated at the junction of the *α*- and *β*-tubulin subunits.^11^ Binding at this juncture inhibits tubulin from adopting the “linear” conformation needed for microtubule assembly. This inhibits polymerization by complicating molecular aggregation.^12,13^

Targeting the CBS is particularly advantageous, as these inhibitors exhibit poor substrate characteristics for the P-glycoprotein (P-gp) efflux pump, thereby allowing them to remain effective against MDR cancer cells.^14,15^ Furthermore, some colchicine-site drugs exhibit substantial vascular-disrupting properties, directly targeting established tumor vasculature.^16^ Colchicine, the primary alkaloid, is highly potent; however, its narrow therapeutic index and notable systemic toxicity have prompted the development of novel synthetic analogs that retain efficacy while minimizing off-target effects.^17^

The intrinsic apoptotic pathway regulates the transition from mitotic arrest to cell death. The Bcl-2 protein family is integral to this process.^18,19^ This “mitochondrial rheostat” maintains a precise balance between pro-apoptotic proteins, such as Bax, and anti-apoptotic proteins, such as Bcl-2. When this equilibrium shifts in favor of Bax, mitochondrial outer membrane permeabilization (MOMP) occurs, allowing cytochrome c to escape and activating Caspase-9.^20^ This initiator caspase subsequently activates the executioner caspase-3, initiating the final phases of cell degradation. Moreover, growing evidence suggests that potent tubulin inhibitors may activate the extrinsic pathway *via* Caspase-8, either by directly activating death receptors or through intricate pathway interactions.^21,22^

Thiazole-based derivatives have emerged as effective antitubulin drugs because the thiazole ring acts as a stiff bioisosteres for the double bond found in natural inhibitors such as Combretastatin A-4 (CA-4). These compounds frequently bind to tubulin's colchicine-binding site, blocking microtubule polymerization, slowing cell division during the G2/M phase, and killing cancer cells.^23,24^

**Fig. 1:**
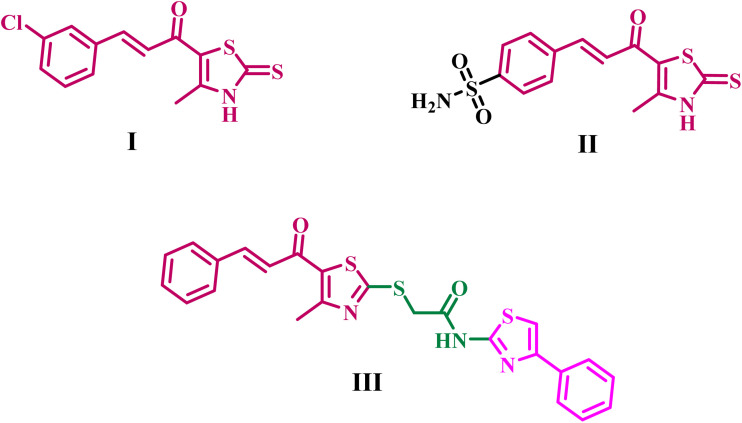
Structures of representative thiazole-based tubulin polymerization inhibitors, I–III.

Hashem *et al.*^23^ described a series of thiazole-privileged chalcones that impede tubulin polymerization, designating compound I, Fig. 1, as the most significant candidate. In the NCI single-dose screen, compound I showed a low mean growth percentage (22.13), indicating potent antiproliferative activity. Five-dose testing across the panel showed low micromolar growth inhibition, with GI_50_ values ranging from 1.55 to 2.95 µM against OVCAR-3 and MDA-MB-468. Compound I reduced tubulin polymerization with an IC_50_ of 7.78 µM (CA-4: 4.93 µM).

Khasawneh *et al.*^25^ enhanced this scaffold by integrating a *para*-sulfamoyl group to develop a dual tubulin/CA IX inhibitor, compound II (Fig. 1). Compound II exhibited significant cytotoxicity against HT-29 cells (IC_50_ = 0.98 µM) while demonstrating favorable selectivity for normal WI-38 fibroblasts (IC_50_ = 44.06 µM), and it inhibited tubulin polymerization (IC_50_ = 2.72 µM, comparable to CA-4 at 2.97 µM). Significantly, it effectively suppressed CA IX (IC_50_ = 0.021 µM) and triggered apoptosis, as evidenced by elevated p53 and Bax levels, decreased Bcl-2 levels, and activation of caspase-3 and caspase-9.

In our latest study,^26^ we identified thiazole-2-acetamide derivatives as inhibitors of tubulin polymerization, with compound III (Fig. 1) demonstrating the highest activity. Compound III reduced tubulin polymerization with an IC_50_ of 2.69 µM and exhibited antiproliferative action in various cancer cell lines, with an average GI_50_ of approximately 6.0 µM. The series had a favorable profile against normal cells, maintaining cell viability above about 85% at 50 µM. In contrast, mechanistic experiments indicated that apoptosis was induced by elevated Bax and caspase-3/9 levels, alongside concurrent downregulation of Bcl-2.

**Fig. 2:**
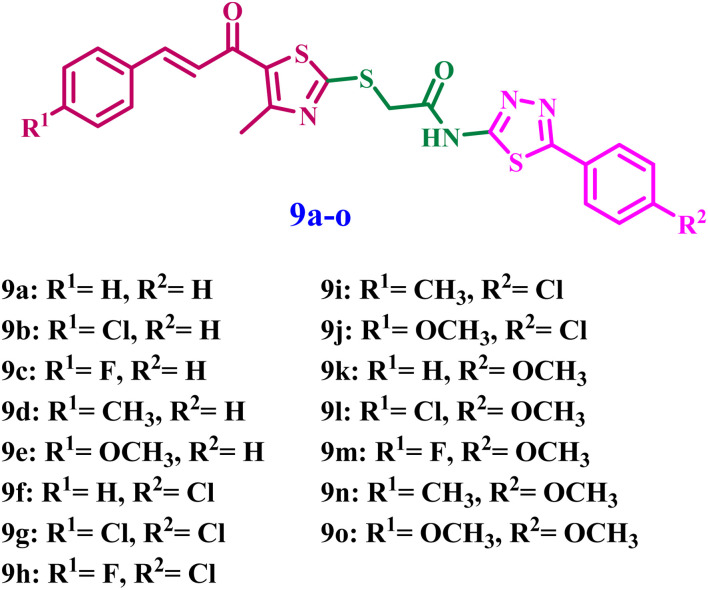
Structures of new compounds 9a–o.

Inspired by prior data and in furtherance of our objective of developing effective tubulin polymerization inhibitors with improved antiproliferative efficacy,^26–29^ we present the synthesis of novel thiazole-thiadiazole derivatives (9a–o) (Fig. 2) for evaluation as antiproliferative agents targeting the colchicine-binding site in tubulin. The novel 9a–o compounds were assessed for their antitubulin activity, and the most effective derivatives were further examined for antiproliferative activity against a selection of cancer cell lines that express tubulin. The concentrations of initiator caspases (Caspase-8 and Caspase-9) and the executioner caspase (Caspase-3) were analyzed to determine the extent of apoptosis. The expression levels of the mitochondrial regulatory proteins Bax and Bcl-2 were analyzed to determine the significance of the intrinsic apoptotic pathway. Subsequently, docking analysis and ADMET investigation followed.

The rational design of the target compounds focused on developing a structurally stable mimic of combretastatin A-4 (CA-4), a strong tubulin inhibitor whose clinical application is frequently hindered by the metabolic isomerization of its *cis*-olefinic bridge. To resolve this, we substituted the unstable ethene linker with a rigid thiazole core. This heterocyclic framework serves as a structural “anchor,” keeping the aromatic substituents in a bioactive orientation that emulates the *cis*-conformation of CA-4 while improving chemical stability. The incorporation of a chalcone-like pharmacophore, recognized for its efficient occupation of the colchicine binding site by aligning its aromatic rings within the deep hydrophobic cavities of the β-tubulin subunit.

Further optimization entailed the integration of a phenyl-1,3,4-thiadiazole terminal moiety as a bioisosteres for the 3,4,5-trimethoxyphenyl A-ring of CA-4. This modification preserves essential hydrophobic interactions while incorporating nitrogen and sulfur heteroatoms that can establish supplementary hydrogen bonds with critical residues in the binding pocket. Finally, the thioacetamide linker connecting these heterocyclic systems imparts the requisite conformational flexibility, enabling the molecule to “thread” into the α/β-tubulin interface, which may augment binding affinity through a synthesis of conventional hydrophobic anchoring and improved polar interactions.

## 2. Experimental

### 2.1. Chemistry

General details: refer to Appendix A (SI).

3-Chloroacetylacetone (2) (ref. 30), 1-(2-mercapto-4-methylthiazol-5-yl)ethan-1-one and (4a–e),^23^ (6a–c), and (7a–c)^31^ were synthesized according to reported procedures.

#### 2.1.1. Synthesis of the target compounds 8a–o

A mixture of thiazole chalcones (4a–e), acylated thiadiazoles (7a–c), anhydrous Na_2_CO_3_, and NaI in acetone was subjected to reflux for 4 hours. The reaction mixture was allowed to cool to room temperature, after which the precipitate was filtered, washed with cold acetone and distilled water, dried, and recrystallized from acetonitrile.

##### 2.1.1.1. 2-((5-Cinnamoyl-4-methylthiazol-2-yl)thio)-*N*-(5-phenyl-1,3,4-thiadiazol-2-yl) acetamide (9a)

Yellow powder; 0.421 g, 88% yield; mp 244–246 °C; ^1^H NMR (500 MHz, DMSO-d_6_) *δ* 13.07 (s, 1H, NH), 7.91 (dd, *J* = 6.5, 2.8 Hz, 2H, Ar–H), 7.76 (dd, *J* = 6.3, 2.6 Hz, 2H, Ar–H), 7.64 (d, *J* = 15.6 Hz, 1H, CH=CH), 7.52–7.46 (m, 3H, Ar–H), 7.45–7.40 (m, 3H, Ar–H), 7.33 (d, *J* = 15.5 Hz, 1H, CH=CH), 4.44 (s, 2H, CH_2_), 2.61 (s, 3H, CH_3_); ^13^C NMR (120 MHz, DMSO-d_6_) *δ* 182.07, 168.26, 166.72, 162.72, 158.84, 158.27, 144.26, 134.65, 132.80, 131.43, 131.24, 130.56, 129.91, 129.54, 129.34, 127.48, 124.82, 37.23, 18.81; anal. calcd. For C_23_H_18_N_4_O_2_S_3_: C, 57.72%; H, 3.79%; N, 11.71%. Found: C, 57.52%; H, 3.98%; N, 11.53%.

##### 2.1.1.2. (*E*)-2-((5-(3-(4-Chlorophenyl)acryloyl)-4-methylthiazol-2-yl)thio)-*N*-(5-phenyl-1,3,4-thiadiazol-2-yl)acetamide (9b)

White powder; 0.416 g, 81% yield; mp 257–260 °C; ^1^H NMR (500 MHz, DMSO-d_6_) *δ* 13.08 (s, 1H, NH), 7.90 (s, 2H, Ar–H), 7.82–7.74 (m, 2H, Ar–H), 7.62 (d, *J* = 14.8, 1H, CH=CH), 7.53–7.42 (m, 5H, Ar–H), 7.34 (d, *J* = 15.4, 1H, CH=CH), 4.45 (s, 2H, CH_2_), 2.60 (s, 3H, CH_3_); ^13^C NMR (120 MHz, DMSO-d_6_) *δ* 181.91, 168.40, 166.69, 162.71, 158.82, 158.43, 142.77, 135.90, 133.62, 132.71, 131.23, 131.04, 130.55, 129.90, 129.56, 127.48, 125.49, 37.23, 18.83; anal. calcd. For C_23_H_17_ClN_4_O_2_S_3_: C, 53.85%; H, 3.34%; N, 10.92%. Found: C, 53.94%; H, 3.26%; N, 10.74%.

##### 2.1.1.3. (*E*)-2-((5-(3-(4-Fluorophenyl)acryloyl)-4-methylthiazol-2-yl)thio)-*N*-(5-phenyl-1,3,4-thiadiazol-2-yl)acetamide (9c)

Yellow powder; 0.447 g, 90% yield; mp 239–241 °C; ^1^H NMR (500 MHz, DMSO-d_6_) *δ* 13.07 (s, 1H, NH), 7.91 (dd, *J* = 6.4, 2.3 Hz, 2H, Ar–H), 7.87–7.83 (m, 2H, Ar–H), 7.65 (d, *J* = 15.5 Hz, 1H, CH=CH), 7.52–7.47 (m, 3H, Ar–H), 7.30 (d, *J* = 15.6 Hz, 1H, CH=CH), 7.26 (t, *J* = 8.8 Hz, 2H, Ar–H), 4.44 (s, 2H, CH_2_), 2.61 (s, 3H, CH_3_); ^13^C NMR (120 MHz, DMSO-d_6_) *δ* 182.00, 168.32, 166.68, 162.71, 161.95, 158.83, 158.25, 143.02, 134.62, 132.78, 131.43, 131.22, 130.56, 129.91, 129.54, 127.48, 124.81, 116.65, 116.48, 37.23, 18.80; anal. calcd. For C_23_H_17_FN_4_O_2_S_3_: C, 55.63%; H, 3.45%; N, 11.28%. Found: C, 55.88%; H, 3.25%; N, 11.07%.

##### 2.1.1.4. (*E*)-2-((4-Methyl-5-(3-(*p*-tolyl)acryloyl)thiazol-2-yl)thio)-*N*-(5-phenyl-1,3,4-thiadiazol-2-yl)acetamide (9d)

Yellow powder; 0.419 g, 85% yield; mp 268–271 °C; ^1^H NMR (500 MHz, DMSO-d_6_) *δ* 13.08 (s, 1H, NH), 7.94–7.86 (m, 2H, Ar–H), 7.67–7.57 (m, 3H, Ar–H & CH=CH), 7.49 (s, 3H, Ar–H), 7.26 (d, *J* = 15.5 Hz, 1H, CH=CH), 7.22 (d, *J* = 7.3 Hz, 2H, Ar–H), 4.44 (s, 2H, CH_2_), 2.60 (s, 3H, CH_3_), 2.30 (s, 3H, CH_3_); ^13^C NMR (120 MHz, DMSO-d_6_) *δ* 181.98, 168.03, 166.70, 162.71, 158.83, 158.11, 144.37, 141.61, 132.84, 131.93, 131.23, 130.56, 130.16, 129.89, 129.38, 127.48, 123.73, 37.23, 21.64, 18.78; anal. calcd. For C_24_H_20_N_4_O_2_S_3_: C, 58.52%; H, 4.09%; N, 11.37%. Found: C, 58.33%; H, 3.99%; N, 11.55%.

##### 2.1.1.5. (*E*)-2-((5-(3-(4-Methoxyphenyl)acryloyl)-4-methylthiazol-2-yl)thio)-*N*-(5-phenyl-1,3,4-thiadiazol-2-yl)acetamide (9e)

Yellowish white powder; 0.402 g, 79% yield; mp 250–252 °C; ^1^H NMR (500 MHz, DMSO-d_6_) *δ* 13.07 (s, 1H, NH), 7.95–7.87 (m, 2H, Ar–H), 7.73 (d, *J* = 8.7 Hz, 2H, Ar–H), 7.62 (d, *J* = 15.4 Hz, 1H, CH=CH), 7.53–7.46 (m, 3H, Ar–H), 7.19 (d, *J* = 15.4 Hz, 1H, CH=CH), 6.97 (d, *J* = 8.7 Hz, 2H, Ar–H), 4.44 (s, 2H, CH_2_), 3.78 (s, 3H, OCH_3_), 2.60 (s, 3H, CH_3_); ^13^C NMR (120 MHz, DMSO-d_6_) *δ* 181.89, 167.74, 166.71, 162.72, 162.14, 158.82, 157.84, 144.36, 132.97, 131.33, 131.23, 130.56, 129.90, 127.48, 127.25, 122.22, 115.04, 55.93, 37.22, 18.75; anal. calcd. For C_24_H_20_N_4_O_3_S_3_: C, 56.67%; H, 3.96%; N, 11.02%. Found: C, 56.53%; H, 4.04%; N, 10.80%.

##### 2.1.1.6. *N*-(5-(4-Chlorophenyl)-1,3,4-thiadiazol-2-yl)-2-((5-cinnamoyl-4-methylthiazol-2-yl)thio)acetamide (9f)

Yellow powder; 0.446 g, 87% yield; mp 275–278 °C; ^1^H NMR (500 MHz, DMSO-d_6_) *δ* 13.11 (s, 1H, NH), 7.96–7.89 (m, 2H, Ar–H), 7.76 (s, 2H, Ar–H), 7.65 (dd, *J* = 15.6, 1H, CH=CH), 7.59–7.52 (m, 2H, Ar–H), 7.43 (s, 3H, Ar–H), 7.34 (dd, *J* = 15.4, 1H, CH=CH), 4.45 (s, 2H, CH_2_), 2.62 (s, 3H, CH_3_); ^13^C NMR (120 MHz, DMSO-d_6_) *δ* 182.05, 168.27, 166.85, 161.49, 159.29, 158.26, 144.25, 135.77, 134.66, 133.85, 132.79, 131.43, 129.94, 129.54, 129.34, 129.14, 124.82, 37.30, 18.80; anal. calcd. For C_23_H_17_ClN_4_O_2_S_3_: C, 53.85%; H, 3.34%; N, 10.92%. Found: C, 53.69%; H, 3.49%; N, 11.14%.

##### 2.1.1.7. (*E*)-*N*-(5-(4-Chlorophenyl)-1,3,4-thiadiazol-2-yl)-2-((5-(3-(4-chlorophenyl)acryloyl)-4-methylthiazol-2-yl)thio)acetamide (9g)

White powder; 0.416 g, 76% yield; mp 262–264 °C; ^1^H NMR (500 MHz, DMSO-d_6_) *δ* 13.10 (s, 1H, NH), 7.92 (d, *J* = 8.5 Hz, 2H, Ar–H), 7.79 (d, *J* = 8.3 Hz, 2H, Ar–H), 7.62 (d, *J* = 15.5 Hz, 1H, CH=CH), 7.55 (d, *J* = 8.4 Hz, 2H, Ar–H), 7.47 (d, *J* = 8.4 Hz, 2H, Ar–H), 7.33 (d, *J* = 15.5 Hz, 1H, CH=CH), 4.44 (s, 2H, CH_2_), 2.60 (s, 3H, CH_3_); ^13^C NMR (120 MHz, DMSO-d_6_) *δ* 181.91, 168.35, 166.76, 161.56, 159.14, 158.41, 142.77, 135.90, 135.80, 133.63, 132.71, 131.03, 129.94, 129.55, 129.43, 129.14, 125.50, 37.24, 18.81; anal. calcd. For C_23_H_16_Cl_2_N_4_O_2_S_3_: C, 50.46%; H, 2.95%; N, 10.23%. Found: C, 50.31%; H, 3.03%; N, 10.34%.

##### 2.1.1.8. (*E*)-*N*-(5-(4-Chlorophenyl)-1,3,4-thiadiazol-2-yl)-2-((5-(3-(4-fluorophenyl)acryloyl)-4-methylthiazol-2-yl)thio)acetamide (9h)

Yellow powder; 0.425 g, 80% yield; mp 246–249 °C; ^1^H NMR (500 MHz, DMSO-d_6_) *δ* 13.13 (s, 1H, NH), 7.92 (d, *J* = 8.1 Hz, 2H, Ar–H), 7.88–7.81 (m, 2H, Ar–H), 7.64 (d, *J* = 15.5 Hz, 1H, CH=CH), 7.55 (d, *J* = 8.3 Hz, 2H, Ar–H), 7.33–7.22 (m, 3H, Ar–H & CH=CH), 4.43 (s, 2H, CH_2_), 2.60 (s, 3H, CH_3_); ^13^C NMR (120 MHz, DMSO-d_6_) *δ* 181.94, 168.20, 166.75, 161.57, 159.08, 158.27, 143.05, 135.80, 132.77, 131.79, 131.35, 129.93, 129.42, 129.14, 124.66, 116.65, 116.48, 37.22, 18.80; anal. calcd. For C_23_H_16_ClFN_4_O_2_S_3_: C, 52.02%; H, 3.04%; N, 10.55%. Found: C, 51.79%; H, 2.96%; N, 10.46%.

##### 2.1.1.9. (*E*)-*N*-(5-(4-Chlorophenyl)-1,3,4-thiadiazol-2-yl)-2-((4-methyl-5-(3-(*p*-tolyl)acryloyl) thiazol-2-yl)thio)acetamide (9i)

Yellow powder; 0.437 g, 83% yield; mp 271–273 °C; ^1^H NMR (500 MHz, DMSO-d_6_) *δ* 13.09 (s, 1H, NH), 7.93 (d, *J* = 6.5 Hz, 2H, Ar–H), 7.66–7.57 (m, 3H, Ar–H & CH=CH), 7.55 (d, *J* = 6.6 Hz, 2H, Ar–H), 7.29–7.20 (m, 3H, Ar–H & CH=CH), 4.44 (s, 2H, CH_2_), 2.60 (s, 3H, CH_3_), 2.31 (s, 3H, Ar-CH_3_); ^13^C NMR (120 MHz, DMSO-d_6_) *δ* 181.98, 167.99, 166.40, 161.58, 158.10, 156.85, 144.37, 141.62, 135.80, 132.84, 131.92, 130.16, 129.95, 129.40, 129.16, 123.72, 111.11, 37.21, 21.64, 18.83; anal. calcd. For C_24_H_19_ClN_4_O_2_S_3_: C, 54.69%; H, 3.63%; N, 10.63%. Found: C, 54.61%; H, 3.49%; N, 10.85%.

##### 2.1.1.10. (*E*)-*N*-(5-(4-Chlorophenyl)-1,3,4-thiadiazol-2-yl)-2-((5-(3-(4-methoxyphenyl) acryloyl)-4-methylthiazol-2-yl)thio)acetamide (9j)

White powder; 0.402 g, 74% yield; mp 237–239 °C; ^1^H NMR (500 MHz, DMSO-d_6_) *δ* 13.11 (s, 1H, NH), 7.93 (d, *J* = 7.6 Hz, 2H, Ar–H), 7.72 (d, *J* = 7.5 Hz, 2H, Ar–H), 7.61 (d, *J* = 15.4 Hz, 1H, CH=CH), 7.55 (d, *J* = 7.3 Hz, 2H, Ar–H), 7.18 (d, *J* = 15.5 Hz, 1H, CH=CH), 6.97 (d, *J* = 7.5 Hz, 2H, Ar–H), 4.43 (s, 2H, CH_2_), 3.78 (s, 3H, OCH_3_), 2.59 (s, 3H, CH_3_); ^13^C NMR (120 MHz, DMSO-d_6_) *δ* 181.88, 167.69, 166.80, 162.14, 161.58, 159.10, 157.83, 144.36, 135.80, 132.98, 131.33, 129.94, 129.43, 129.15, 127.25, 122.21, 115.04, 55.93, 37.21, 18.73; anal. calcd. For C_24_H_19_ClN_4_O_3_S_3_: C, 53.08%; H, 3.53%; N, 10.32%. Found: C, 52.99%; H, 3.77%; N, 10.15%.

##### 2.1.1.11. 2-((5-Cinnamoyl-4-methylthiazol-2-yl)thio)-*N*-(5-(4-methoxyphenyl)-1,3,4-thiadiazol-2-yl)acetamide (9k)

Yellow powder; 0.463 g, 91% yield; mp 277–280 °C; ^1^H NMR (500 MHz, DMSO-d_6_) *δ* 12.99 (s, 1H, NH), 7.84 (d, *J* = 8.6 Hz, 2H, Ar–H), 7.77–7.72 (m, 2H, Ar–H), 7.64 (d, *J* = 15.5 Hz, 1H, CH=CH), 7.45–7.39 (m, 3H, Ar–H), 7.32 (d, *J* = 15.5 Hz, 1H, CH=CH), 7.03 (d, *J* = 8.6 Hz, 2H, Ar–H), 4.43 (s, 2H, CH_2_), 3.78 (s, 3H, OCH_3_), 2.61 (s, 3H, CH_3_); ^13^C NMR (120 MHz, DMSO-d_6_) *δ* 182.03, 168.31, 166.56, 162.52, 161.66, 158.29, 158.18, 144.24, 134.64, 132.78, 131.43, 129.54, 129.33, 129.04, 124.78, 123.08, 115.28, 55.95, 37.24, 18.81; anal. calcd. For C_24_H_20_N_4_O_3_S_3_: C, 56.67%; H, 3.96%; N, 11.02%. Found: C, 56.54%; H, 3.75%; N, 11.21%.

##### 2.1.1.12. (*E*)-2-((5-(3-(4-Chlorophenyl)acryloyl)-4-methylthiazol-2-yl)thio)-*N*-(5-(4-methoxy phenyl)-1,3,4-thiadiazol-2-yl)acetamide (9l)

Yellow powder; 0.424 g, 78% yield; mp 255–257 °C; ^1^H NMR (500 MHz, DMSO-d_6_) *δ* 12.99 (s, 1H, NH), 7.84 (d, *J* = 8.8 Hz, 2H, Ar–H), 7.80 (d, *J* = 8.4 Hz, 2H, Ar–H), 7.63 (d, *J* = 15.5 Hz, 1H, CH=CH), 7.47 (d, *J* = 8.4 Hz, 2H, Ar–H), 7.34 (d, *J* = 15.5 Hz, 1H, CH=CH), 7.04 (d, *J* = 8.8 Hz, 2H, Ar–H), 4.42 (s, 2H, CH_2_), 3.79 (s, 3H, OCH_3_), 2.61 (s, 3H, CH_3_); ^13^C NMR (120 MHz, DMSO-d_6_) *δ* 181.91, 168.45, 166.53, 162.52, 161.66, 158.44, 158.14, 142.77, 135.90, 133.62, 132.70, 131.05, 129.56, 129.05, 125.48, 123.07, 115.28, 55.95, 37.22, 18.82; anal. calcd. For C_24_H_19_ClN_4_O_3_S_3_: C, 53.08%; H, 3.53%; N, 10.32%. Found: C, 53.21%; H, 3.66%; N, 10.14%.

##### 2.1.1.13. (*E*)-2-((5-(3-(4-fluorophenyl)acryloyl)-4-methylthiazol-2-yl)thio)-*N*-(5-(4-methoxy phenyl)-1,3,4-thiadiazol-2-yl)acetamide (9m)

White powder; 0.432 g, 82% yield; mp 263–266 °C; ^1^H NMR (500 MHz, DMSO-d_6_) *δ* 12.99 (s, 1H, NH), 7.88–7.81 (m, 4H, Ar–H), 7.65 (d, *J* = 15.5 Hz, 1H, CH=CH), 7.30 (d, *J* = 15.6 Hz, 1H, CH=CH), 7.28–7.24 (m, 2H, Ar–H), 7.04 (d, *J* = 8.7 Hz, 2H, Ar–H), 4.43 (s, 2H, CH_2_), 3.79 (s, 3H, OCH_3_), 2.61 (s, 3H, CH_3_); ^13^C NMR (120 MHz, DMSO-d_6_) *δ* 181.97, 168.26, 163.08, 162.54, 161.67, 158.27, 143.04, 132.76, 131.80, 131.73, 131.36, 129.05, 124.69, 123.09, 116.65, 116.48, 115.29, 55.96, 37.23, 18.81; anal. calcd. For C_24_H_19_FN_4_O_3_S_3_: C, 54.74%; H, 3.64%; N, 10.64%. Found: C, 54.50%; H, 3.47%; N, 10.89%.

##### 2.1.1.14. (*E*)-*N*-(5-(4-Methoxyphenyl)-1,3,4-thiadiazol-2-yl)-2-((4-methyl-5-(3-(*p*-tolyl) acryloyl)thiazol-2-yl)thio)acetamide (9n)

Yellowish white powder; 0.449 g, 86% yield; mp 248–250 °C; ^1^H NMR (500 MHz, DMSO-d_6_) *δ* 12.99 (s, 1H, NH), 7.87–7.81 (m, 2H, Ar–H), 7.68–7.59 (m, 3H, Ar–H), 7.27 (d, *J* = 15.7 Hz, 1H, CH=CH), 7.23 (d, *J* = 5.9 Hz, 2H, Ar–H), 7.07–7.02 (m, 2H, Ar–H), 4.42 (s, 2H, CH_2_), 3.79 (s, 3H, OCH_3_), 2.61 (s, 3H, CH_3_), 2.31 (s, 3H, Ar–CH_3_); ^13^C NMR (120 MHz, DMSO-d_6_) *δ* 181.96, 169.40, 168.11, 166.56, 162.52, 161.65, 158.12, 144.37, 141.62, 132.83, 131.91, 130.16, 129.40, 129.05, 123.70, 123.07, 115.28, 55.94, 37.22, 21.65, 18.79; anal. calcd. For C_25_H_22_N_4_O_3_S_3_: C, 57.45%; H, 4.24%; N, 10.72%. Found: C, 57.37%; H, 4.02%; N, 10.92%.

##### 2.1.1.15. (*E*)-*N*-(5-(4-Methoxyphenyl)-1,3,4-thiadiazol-2-yl)-2-((5-(3-(4-methoxyphenyl) acryloyl)-4-methylthiazol-2-yl)thio)acetamide (9o)

White powder; 0.404 g, 75% yield; mp 240–242 °C; ^1^H NMR (500 MHz, DMSO-d_6_) *δ* 13.05 (s, 1H, NH), 7.87 (d, *J* = 8.8 Hz, 2H, Ar–H), 7.73 (d, *J* = 8.7 Hz, 2H, Ar–H), 7.61 (d, *J* = 15.5 Hz, 1H, CH=CH), 7.21 (d, *J* = 15.5 Hz, 1H, CH=CH), 7.04 (d, *J* = 8.8 Hz, 2H, Ar–H), 6.97 (d, *J* = 8.7 Hz, 2H, Ar–H), 4.43 (s, 2H, CH_2_), 3.79 (s, 6H, 2×OCH_3_), 2.60 (s, 3H, CH_3_); ^13^C NMR (120 MHz, DMSO-d_6_) *δ* 181.90, 167.72, 166.80, 162.52, 162.14, 161.66, 159.10, 157.83, 144.35, 132.98, 131.33, 130.56, 129.90, 129.54, 127.25, 122.22, 115.28, 115.04, 55.94, 37.22, 18.74; anal. calcd. For C_25_H_22_N_4_O_4_S_3_: C, 55.75%; H, 4.12%; N, 10.40%. Found: C, 55.83%; H, 4.22%; N, 10.30%.

### 2.2. Biology

#### 2.2.1. Cell viability assay

Using the human mammary gland epithelial normal cell line (MCF-10A), the effects of compounds 9a–o on cell viability were evaluated. Following a four-days incubation with MCF-10A cells, the cell viability of compounds 9a–o was evaluated using the MTT assay.^32,33^ For further details, check Appendix A.

#### 2.2.2. Tubulin polymerization assay

Utilizing the Tubulin Polymerization Assay Kit (Cytoskeleton Inc., Denver, CO, USA), the effects of compounds 9a–o on tubulin polymerization were examined^27,28^ in accordance with the supplier's guidelines. Appendix A (SI) contains the specifics.

#### 2.2.3. Antiproliferative assay

The MTT test^34^ was used to assess the antiproliferative efficacy of novel compounds 9e, 9g, 9i, 9k, and 9m against three cancer cell lines: A549 (Human Lung Adenocarcinoma), HeLa (Human Cervical Cancer), and HCT 116 (Human Colorectal Adenocarcinoma). All cell lines were acquired from ATCC (American Type Culture Collection) *via* the Holding Company for Biological Products and Vaccines (VACSERA) in Cairo, Egypt. In this investigation, CA-4 served as the control. Refer to Appendix A for more experimental details.

#### 2.2.4. Apoptotic markers assays

In the HeLa cervical cancer cell line, compound 9k was evaluated for its capacity to activate caspase-3 and caspase-9, induce Bax activation, and down-regulate the anti-apoptotic protein Bcl-2.^35^ Additional information is available in Appendix A.

## 3. Results and discussion

### 3.1. Chemistry

**Scheme 1:**
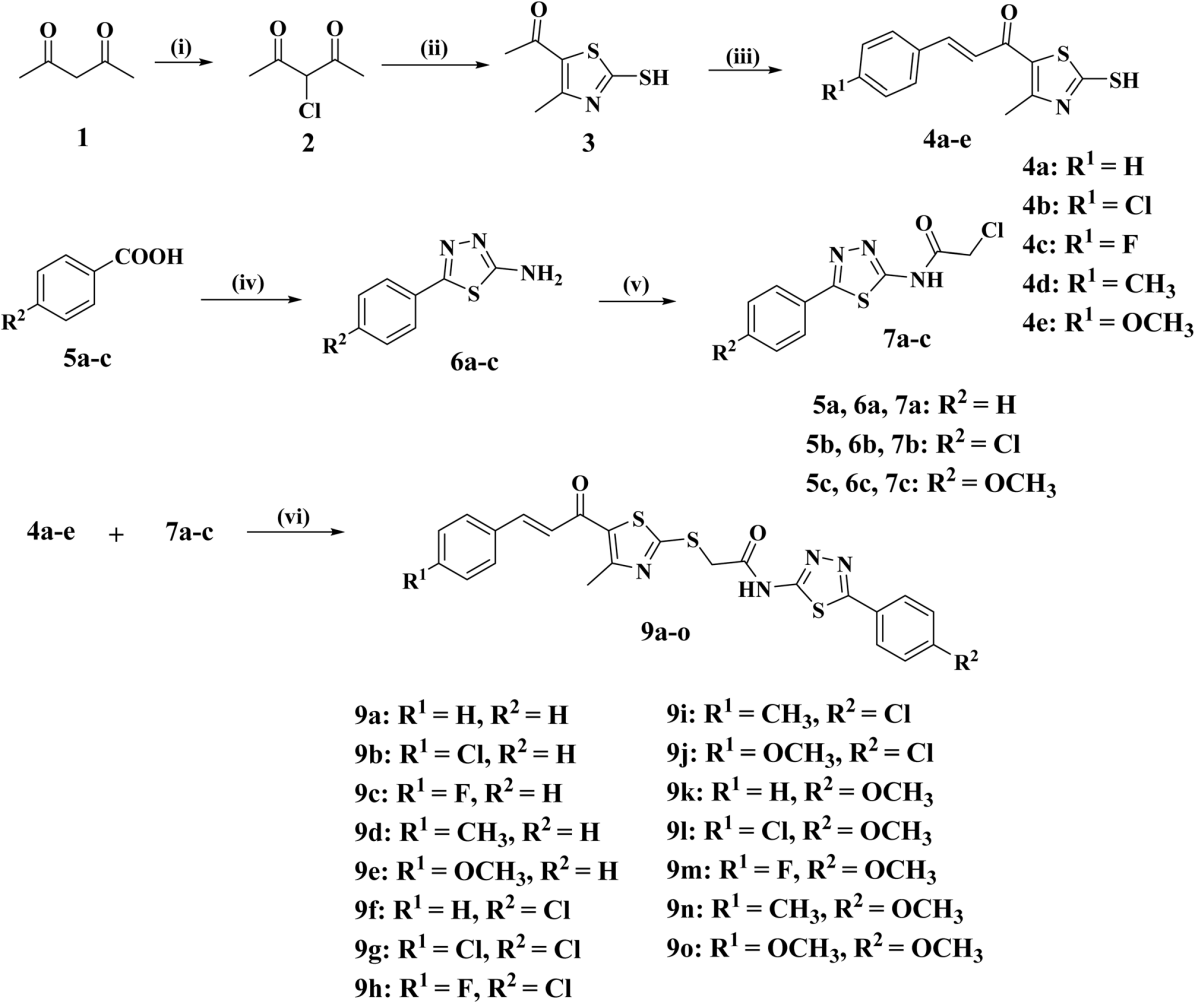
Synthesis of the target compounds 9a–o. Reagents and Conditions: (i) SO_2_Cl_2_, toluene, 0 °C, 12 h; (ii) NH_3_, CS_2_, EtOH, RT, 6 h; (iii) appropriate aromatic aldehyde, 60% NaOH, EtOH, 0 °C, 18 h.; (iv) thiosemicarbazide, POCl_3_, reflux, 5 h.; (v) ClCH_2_COCl, benzene, reflux, 3–5 h.; (vi) Na_2_CO_3_, NaI, acetone, RT, 6 h.

As outlined in Scheme 1, the target compounds 9a–o were synthesized *via* a convergent synthetic route involving two key intermediates: mercapto-thiazole chalcones (4a–e) and chloroacetamido-thiadiazoles (7a–c). The thiazole core was synthesized through heterocyclization of 3-chloro acetylacetone^30^ with carbon disulfide and ammonia to yield the thiazole intermediate 3, which subsequently underwent base-catalyzed Claisen–Schmidt condensation with substituted aldehydes to afford the corresponding chalcones (4a–e).^23^

Concurrently, substituted benzoic acids 5a–c were converted into 1,3,4-thiadiazole amines (6a–c) *via* condensation with thiosemicarbazide followed by POCl_3_-mediated cyclodehydration. These were then acylated with chloroacetyl chloride to give the electrophilic chloroacetamides (7a–c).^31^ Final coupling with chalcones (4a–e) was achieved under mild basic conditions through an *S*-alkylation reaction, affording the final compounds 9a–o.

Compounds 9a–o were characterized by ^1^H NMR, ^13^C NMR, and elemental analysis (C, H, N), confirming formation of the targeted thioether-linked thiazole–chalcone/1,3,4-thiadiazole hybrids. In the ^1^H NMR spectra, all derivatives displayed a diagnostic amide NH as a downfield singlet at *δ* 12.99–13.13 (1H, s). The α,β-unsaturated enone fragment consistently appeared as two *trans*-olefinic doublets (CH=**CH**) at *δ* 7.61–7.65 (1H, d, *J* ≈ 14.8–15.6 Hz) and *δ* 7.18–7.34 (1H, d, *J* ≈ 15.4–15.7 Hz), and the large coupling constant supports the *E* configuration across the series. The aromatic protons of the two phenyl rings resonated mainly in the *δ* 6.97–7.96 region. The characteristic methylene signal was observed as a singlet at *δ* 4.42–4.45 (2H, s), while the thiazole methyl group appeared as a sharp singlet at *δ* 2.59–2.62 (3H, s). Compounds containing methoxy groups showed an extra singlet at 3.78–3.79, while *p*-tolyl derivatives showed an additional singlet at *δ* 2.30–2.31.

In the ^13^C NMR spectra, the chalcone carbonyl was observed in the expected downfield region at *δ* 181.88–182.07, while the acetamide carbonyl (C=O) appeared at *δ* 166.40–169.40. Aliphatic carbons were also diagnostic, such as the methylene moiety, which appeared at *δ* 37.21–37.30, the thiazole methyl at *δ* 18.73–18.83, the methoxy carbon at *δ* 55.93–55.96 (when present), and the *p*-tolyl methyl carbon at *δ* 21.64–21.65 (when present).

As a representative example, compound 9l showed ^1^H and ^13^C signals fully consistent with its substitution pattern. In the ^1^H NMR spectrum, the amide proton appeared at *δ* 12.99, while the enone moiety gave two *trans*-coupled vinylic doublets at *δ* 7.63 and *δ* 7.34, confirming the *E* geometry. The aromatic region exhibited two *para*-disubstituted patterns and appeared as 4 doublets: the *p*-chlorophenyl chalcone ring resonated at *δ* 7.80 and *δ* 7.47, whereas the *p*-methoxyphenyl thiadiazole ring appeared at *δ* 7.84 and *δ* 7.04. The methylene was observed at *δ* 4.42, the methoxy group at *δ* 3.79, and the thiazole methyl at *δ* 2.61. In the ^13^C NMR spectrum, the expected enone carbonyl appeared at *δ* 181.91 and the amide carbonyl at *δ* 168.45. The methoxy carbon appeared at *δ* 55.95, while the methylene and thiazole methyl were observed at *δ* 37.22 and *δ* 18.82, respectively. Taken together, the ^1^H/^13^C features of 9l mirror the general spectral fingerprint of the series and further confirm the structures of 9a–o.

### 3.2. Biology

#### 3.2.1. Cell viability assay

The MCF-10A (human mammary gland epithelial) normal cell line was utilized to study the impact of the novel compounds 9a–o on cell viability. Following a 4 days incubation with MCF-10A cells, the survival of the novel compounds was determined using the MTT assay.^32,33^Table 1 shows that at a concentration of 50 µM, all compounds tested maintained cell viability above 88% and had no lethal effects on normal cells.

**Table 1:** % of Cell viability and IC_50_ values of compounds 9a–o against tubulin^a^

Compound	R_1_	R_2_	Cell viability%	Tubulin inhibition IC_50_ ± SEM (µM)
9a	H	H	90	29.66 ± 1.20
9b	Cl	H	93	6.07 ± 0.25
9c	F	H	91	19.45 ± 1.10
9d	Me	H	90	15.60 ± 0.70
9e	OMe	H	92	3.04 ± 0.11
9f	H	Cl	88	22.67 ± 0.81
9g	Cl	Cl	91	5.33 ± 0.17
9h	F	Cl	88	8.11 ± 0.30
9i	Me	Cl	91	2.71 ± 0.09
9j	OMe	Cl	93	11.60 ± 0.55
9k	H	OMe	90	1.56 ± 0.05
9l	Cl	OMe	89	37.21 ± 1.50
9m	F	OMe	90	5.74 ± 0.20
9n	Me	OMe	89	26.40 ± 1.05
9o	OMe	OMe	90	32.15 ± 1.20
CA-4	—	—	—	2.80 ± 0.10

a –: not applicable.

#### 3.2.2. Tubulin inhibitory assay

The effects of all newly developed compounds 9a–o as inhibitors of tubulin polymerization, using CA-4 as a reference medication,^27,28^ are detailed in Table 1. Compounds 9a–o exhibited potent anti-tubulin activity, with IC_50_ values ranging from 1.56 to 37.21 µM, compared with the standard CA-4 (IC_50_ = 2.80 µM). Compounds 9b, 9e, 9g, 9i, 9k, and 9m exhibited the most pronounced anti-tubulin activity, with IC_50_ values ranging from 1.56 to 6.07 µM. Compounds 9i and 9k exhibited the highest potency among the derivatives, with IC_50_ values of 2.71 and 1.56 µM, respectively, exceeding that of the reference drug CA-4 (IC_50_ = 2.80 µM).

Compound 9k (R^1^ = H, R^2^ = OMe) had the highest potency as an antitubulin agent, with an IC_50_ value of 1.56 µM, demonstrating double the efficacy of the reference compound CA-4. The anti-tubulin efficacy of compounds 9a–o is markedly influenced by the substitution pattern on the phenyl groups of the chalcone moiety (R^1^) and at the fourth position of the thiadiazole moiety (R^2^). For instance, compounds 9a (R^1^ = R^2^ = H) and 9f (R^1^ = H, R^2^ = Cl) both include an unsubstituted phenyl group on the chalcone moiety, akin to compound 9k, yet possess distinct substituents at the *para* position of the phenyl group inside the thiadiazole moiety. Compounds 9a and 9f exhibited diminished potency relative to 9k, with IC_50_ values of 29.66 µM for 9a and 22.67 µM for 9f, representing 19-fold and 15-fold reductions in potency compared to 9k, respectively. The data indicated that, when the phenyl group of the chalcone moiety is unsubstituted, the methoxy group is the optimal substituent on the phenyl group of the thiadiazole moiety, followed by the chlorine atom. Additionally, when both phenyl groups are unsubstituted, it is detrimental to antitubulin activity.

Conversely, compounds 9l (R^1^ = Cl, R^2^ = OMe), 9m (R^1^ = F, R^2^ = OMe), 9n (R^1^ = Me, R^2^ = OMe), and 9o (R^1^ = OMe, R^2^ = OMe) possess a methoxy group in the *para* position of the phenyl group within the thiadiazole moiety, similar to 9k, but feature varying substituents on the phenyl group of the chalcone moiety (R^1^). Except for 9m, compounds 9l, 9n, and 9o showed a significant reduction in antitubulin activity, with IC_50_ values of 37.21, 26.4, and 32.15 µM, respectively, indicating at least a 17-fold drop in potency relative to 9k. Compound 9m, containing a fluorine atom, showed significant antitubulin activity with an IC_50_ of 5.74 µM, 3.7-fold less effective than 9k. These data indicated that the presence of a methoxy group at the *para* position of the phenyl group in the thiadiazole moiety renders the unsubstituted phenyl group in the chalcone moiety optimum for activity. Substitution with electron-donating groups such as methyl and methoxy, or with electron-withdrawing groups such as chlorine or fluorine, reduces activity, with fluorine ranking second in effect after hydrogen.

Compound 9i (R^1^ = Me, R^2^ = Cl) scored second in antitubulin activity, with an IC_50_ value of 2.71 µM. It was 1.7-fold less effective than compound 9k, but marginally more potent than the standard CA-4. Compounds 9f (R^1^ = H, R^2^ = Cl), 9g (R^1^ = Cl, R^2^ = Cl), 9h (R^1^ = F, R^2^ = Cl), and 9j (R^1^ = OMe, R^2^ = Cl) all possess a chlorine atom as R^2^, similar to 9i, but feature distinct R^1^ substituents. Excluding 9f, compounds 9g, 9h, and 9j exhibited moderate antitubulin activity with IC_50_ values of 5.33, 8.11, and 11.60 µM, respectively. Compound 9f exhibited a significant reduction in its antitubulin activity, with an IC_50_ value of 22.67 µM. The study found that when a chlorine atom is present as an R^2^ substituent within the thiadiazole moiety, the unsubstituted phenyl group in the chalcone moiety has reduced activity. Still, chlorine and fluorine atoms (as R^1^, electron-withdrawing groups) are the best for activity. Ultimately, compound 9o (R^1^ = R^2^ = OMe) had the lowest potency among all synthesised derivatives as an antitubulin agent. It exhibited an IC_50_ of 32.15 µM, indicating a 20-fold reduction in activity compared to 9k, suggesting that the presence of a methoxy group on both phenyl rings is detrimental to activity.

#### 3.2.3. Antiproliferative assay

Compounds 9e, 9g, 9i, 9k, and 9m, the most effective tubulin inhibitors, were subsequently evaluated for their antiproliferative effects against three cancer cell lines: A549 (Human Lung Adenocarcinoma), HeLa (Human Cervical Cancer), and HCT 116 (Human Colorectal Adenocarcinoma), utilizing the MTT assay.^36^CA-4 was used as the reference compound; Table 2 presents the results as IC_50_ values and average IC_50_ values (GI_50_) against the three cancer cell lines for each compound.

**Table 2:** IC_50_ values of compounds 9e, 9g, 9i, 9k, and 9m

Compd	Antiproliferative activity IC_50_ ± SEM (µM)			
	HCT-116	A-549	Hela	GI_50_
9e	8.91 ± 0.06	10.23 ± 0.90	7.72 ± 0.05	8.98
9g	9.64 ± 0.07	11.40 ± 0.95	7.68 ± 0.05	9.57
9i	7.51 ± 0.05	9.72 ± 0.07	8.65 ± 0.06	8.62
9k	7.29 ± 0.05	8.01 ± 0.05	5.62 ± 0.03	6.97
9m	15.22 ± 1.05	11.98 ± 1.01	8.59 ± 0.06	11.93
CA-4	2.35 ± 0.002	1.12 ± 0.001	2.01 ± 0.002	1.82

The outcomes of the *in vitro* antiproliferative assay corresponded with those of the antitubulin assay. Compounds 9e, 9g, 9i, 9k, and 9m demonstrated significant antiproliferative activity, with GI_50_ values ranging from 6.97 to 11.93 µM, compared with the reference medication CA-4, which had a GI_50_ of 1.82 µM across the three cancer cell lines examined. In every instance, the studied compounds were less potent than the reference CA-4 across all cancer cell lines evaluated. Compound 9k (R^1^ = H, R^2^ = OMe), the most effective tubulin inhibitor, exhibited the highest antiproliferative activity with a GI_50_ value of 6.97 µM. It showed a fourfold decrease in potency compared with CA-4 against the cancer cell lines evaluated.

Compound 9k exhibited the highest potency among derivatives against the cervical (HeLa), colorectal (HCT-116), and lung (A-549) cancer cell lines, with IC_50_ values of 5.62, 7.29, and 8.01 µM, respectively, suggesting that cervical cancer cells demonstrate greater sensitivity to the mechanism of action of 9k compared to colon or lung cancer models. The increased sensitivity in HeLa cells could be attributable to their rapid proliferation rate, as tubulin inhibitors frequently exhibit higher toxicity in more rapidly dividing cells.^37^

Compounds 9e (R^1^ = OMe, R^2^ = H) and 9i (R^1^ = Me, R^2^ = Cl) exhibited the third and second-highest antiproliferative activity, with GI_50_ values of 8.98 and 8.62 µM, respectively. They exhibited approximately a 1.3-fold reduction in potency relative to 9k. The HeLa (cervical) cancer cell line was the most responsive to compound 9e, while the colorectal (HCT-116) cancer cell line showed the highest sensitivity to compound 9i. Compounds 9g (R^1^ = Cl, R^2^ = Cl) and 9m (R^1^ = Me, R^2^ = OMe) exhibited the lowest antiproliferative efficacy, with GI_50_ values of 9.57 and 11.93 µM, respectively, signifying modest antiproliferative activity, which correlates with antitubulin activity reflected by IC_50_ values of 5.33 and 5.74 µM, respectively, Table 1.

#### 3.2.4. Apoptotic markers assays

Among tubulin inhibitors, Cytochrome c serves as the “messenger of death.” Its release from the mitochondria into the cytoplasm is a hallmark of intrinsic apoptosis (programmed cell death) triggered by microtubule rupture.^38^ Tubulin inhibitors hinder microtubule assembly or disassembly. This makes it impossible for the cell to assemble a functional mitotic spindle.^39^ During the M-phase of the cell cycle, the cell cannot move forward. When a cell remains in mitosis for an extended period, it activates pro-apoptotic proteins, including Bax.^40^ These proteins make tiny holes in the outer membrane of the mitochondria. The process is called Mitochondrial Outer Membrane Permeabilization (MOMP). When the mitochondrial membrane is punctured, Cytochrome c, which normally stays in the mitochondria to help make energy, leaks into the cytoplasm.^41^ Cytochrome c binds to a protein called Apaf-1 in the cytoplasm to form a complex called the Apoptosome. The Apoptosome acts as a “trigger” that activates Caspase-9, which then activates Caspase-3. These enzymes act like molecular scissors, cutting DNA and proteins in the cell, leading to the death of cancer cells.^42,43^

Consequently, the capability of compound 9k to function as an activator of Bax, Caspases-3, -8, and -9, as well as a down-regulator of the anti-apoptotic Bcl-2, was examined.

##### 3.2.4.1. Caspase- 3, -8, and -9 activation assay

Compound 9k, the most effective tubulin inhibitor and antiproliferative agent, was evaluated for its activation of caspases-3, -8, and -9 in the HeLa cervical cancer cell line,^44^ with results presented in Table 3. The findings showed that compound 9k had significantly higher caspase-3 protein levels (587 ± 4 pg mL^−1^) than the standard compound, staurosporine (510 ± 4 pg mL^−1^). Compound 9k significantly increased total caspase-3 protein levels in the HeLa cervical cancer cell line, with values nine times higher than those of untreated control cells and above those of the reference drug, staurosporine.

**Table 3:** Caspase-3/8/9 activation of compound 9k against the HeLa cervical cancer cell line

Compound number	Caspase-3		Caspase-8		Caspase-9	
	Conc (pg mL^−1^)	Fold change	Conc (ng mL^−1^)	Fold change	Conc (ng mL^−1^)	Fold change
9k	589 ± 4	9	1.12 ± 0.10	11	21.4 ± 1	21
Staurosporine	510 ± 4	8	1.90 ± 0.10	19	20 ± 1	20
Control	65	1	0.10	1	1	1

Furthermore, activation testing of caspase-8 and -9 indicated that compound 9k markedly increased their levels compared with staurosporine. Compound 9k demonstrated substantial overexpression of caspase-9 (21.4 ng mL^−1^, 21-fold increase), followed by caspase-8 (1.12 ng mL^−1^, 11-fold increase). The data indicate that apoptosis may contribute to the antiproliferative effects of the tested compound, through activation of both intrinsic and extrinsic pathways, with a greater impact on the intrinsic pathway, as evidenced by elevated caspase-9 levels. This suggests that 21-fold activation of Caspase-9 is sufficient to initiate the proteolytic cascade. Although the Caspase-8 route is reduced, the “apoptotic engine” of 9k remains effective, resulting in a higher concentration of active Caspase-3 (589 ± 4 pg mL^−1^). In summary, even with reduced caspase-8 activation, 9k remains more proficient in achieving the final stage of apoptosis.

##### 3.2.4.2. Assay for levels of Bax and Bcl-2

We also investigated the impact of compound 9k on the apoptotic marker Bax and the anti-apoptotic Bcl-2 levels in the HeLa cancer cell line, using staurosporine as a ref. 44. The results are presented in Table 4.

**Table 4:** Bax and Bcl-2 levels of 9k in cervical (HeLa) cell line

Compound number	Bax		Bcl-2	
	Conc (pg mL^−1^)	Fold change	Conc (ng mL^−1^)	Fold reduction
9k	307 ± 5	38	1.05	5
Staurosporine	280 ± 7	35	1.10	5
Control	8	1	5	1

The Bax/Bcl-2 ratio is pivotal in dictating cellular outcomes. Compound 9k significantly shifts the equilibrium towards mortality. Bax is a protein that facilitates apoptosis by creating pores in the outer mitochondrial membrane. A 38-fold increase (307 ± 5 pg mL^−1^) is substantial, exceeding the effect of staurosporine (35-fold). Bcl-2 is an anti-apoptotic protein that generally inhibits Bax activity. Reducing its concentration to 1.05 ng mL^−1^ (a fivefold reduction) reduces apoptosis inhibition.

These findings elucidate the mechanism underlying the previously observed 21-fold elevation in Caspase-9 levels. The inhibition of tubulin (IC_50_ = 1.56 µM) induces mitotic stress. The cell responds by elevating Bax levels and reducing Bcl-2 levels. An elevated Bax/Bcl-2 ratio leads to mitochondrial outer membrane permeabilization (MOMP). Upon the leakage of Cytochrome c, caspase-9 is activated, subsequently activating caspase-3.

### 3.3. *In silico* studies

#### 3.3.1. Molecular docking

**Fig. 3:**
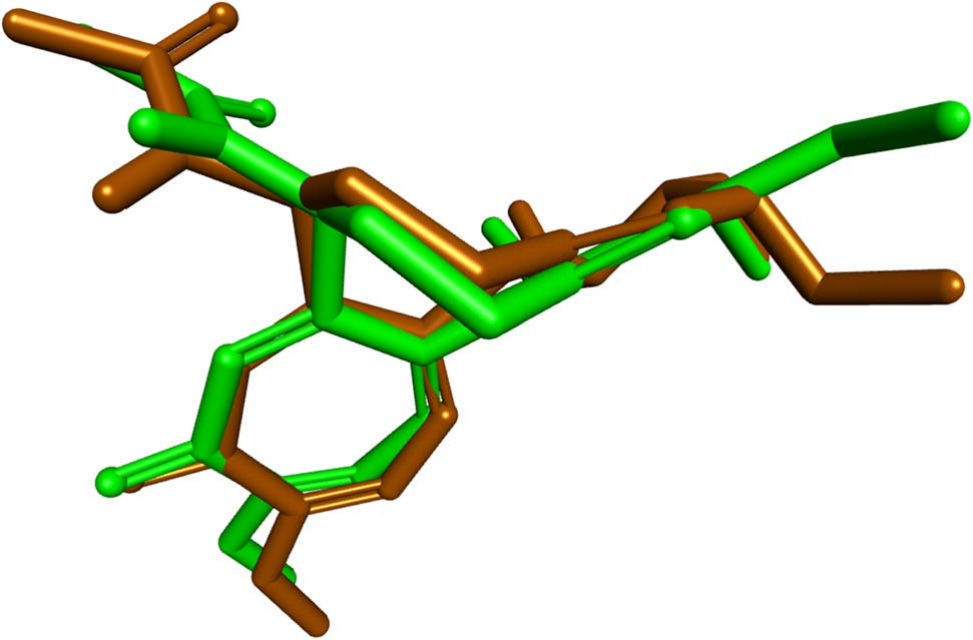
Superimposition of cocrystallized and redocked colchicine in the colchicine binding site of tubulin (RMSD = 0.9278 Å).

Molecular docking was employed as a structure-based approach to rationalize the interaction pattern of the designed tubulin inhibitors within the colchicine binding site and to correlate their calculated binding modes with the observed cytotoxic profile. Using the X-ray crystal structure of tubulin in complex with colchicine (PDB ID: 4O2B)^45^ as the receptor, docking was performed with AutoDock Vina,^46^ and the resulting poses were analyzed using Discovery Studio Visualizer.^47^ Redocking of colchicine afforded a binding affinity of −7.8 kcal mol^−1^, and superimposing the redocked and cocrystallized poses yielded an RMSD of 0.9278 Å (Fig. 3), which is below the accepted 2.0 Å threshold, confirming that the docking protocol reliably reproduces the experimental binding mode.

**Fig. 4:**
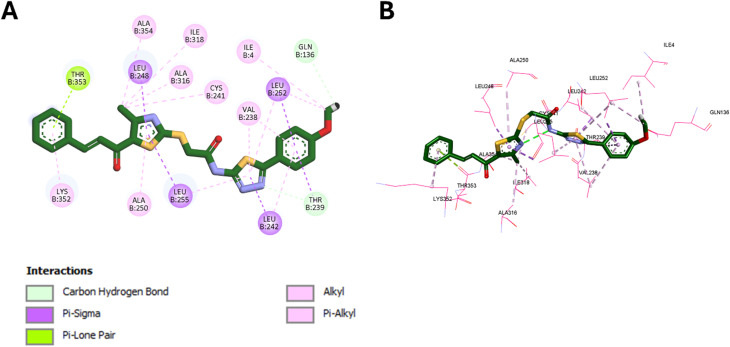
Binding mode of 9k in the CBS of tubulin: (A) 2D diagram; (B) 3D binding pose.

Within this validated setup, docking of the most potent derivative 9k into the colchicine binding site (CBS) revealed a binding affinity of −9.1 kcal mol^−1^, surpassing that of colchicine (−7.8 kcal mol^−1^) and supporting its superior antiproliferative activity. Compound 9k fits deeply into the hydrophobic pocket at the α/β-tubulin interface and establishes a dense network of favorable contacts with key residues (Fig. 4).

The *p*-methoxyphenyl–thiadiazole portion forms a carbon–hydrogen bond with Gln136 and hydrophobic interactions with Ile4, Leu252, Leu242, Thr239, and Val238, residues that define the colchicine pocket and contribute critically to ligand affinity. The thiadiazole and thiazole rings further engage Leu238, Leu248, Leu255, and Ala250 through pi–sigma and pi–alkyl contacts, while the thiazole-methyl group interacts with Ala354, Ile318, Ala316, and Cys241 in the deeper hydrophobic groove. Finally, the terminal chalcone benzene ring interacts with Lys352 at the pocket entrance, helping to stabilize the ligand orientation. Altogether, this interaction pattern, involving hallmark residues such as Leu242, Leu248, Leu252, Leu255, Val238, Ala250, Ala316, Ile318, Ala354, Lys352, and Cys241, is highly consistent with the known pharmacophoric features of colchicine binding site inhibitors and provides a clear structural basis for the marked tubulin-targeted cytotoxicity of compound 9k.

To further address the observed SAR trends and clarify the influence of substitution at R1 and R2, additional comparative docking analyses were performed for compounds 9e, 9i, 9m, and 9l within the same colchicine binding site. The obtained binding modes were generally consistent with the experimental antitubulin data and showed that activity was governed not simply by the number of contacts, but rather by the ability of each analogue to adopt a productive orientation and establish well-positioned polar and hydrophobic interactions within the CBS.

Among the newly examined analogues, 9i displayed the most favorable interaction pattern, in agreement with its high tubulin inhibitory activity. It formed a classical hydrogen bond between the thiadiazole nitrogen and Tyr202, in addition to an extended hydrophobic network involving Leu248, Ala250, Leu255, Ala316, Ala354, Ile318, Leu242, Val238, and Thr239. Moreover, the *p*-chloro substituent on the thiadiazole-linked phenyl ring occupied a lipophilic region and contributed additional favorable contacts with Phe169, Phe20, and Met235, supporting the beneficial effect of the Me/Cl substitution pattern in compound 9i.

Compound 9e also adopted a productive binding mode consistent with its potent activity. A key feature was the formation of a classical hydrogen bond between the chalcone carbonyl and Asn258, while the aromatic scaffold was further stabilized by several hydrophobic interactions with Lys352, Leu248, Leu255, Ala316, Leu242, Leu252, and Thr239. The *p*-methoxy substituent on the chalcone phenyl ring also contributed favorably through hydrophobic interaction with Lys352, indicating that R1 = OMe is advantageous when the thiadiazole-linked phenyl ring is unsubstituted.

In the case of 9m, which showed moderate activity, the docked pose retained several favorable hydrophobic interactions with Leu248, Ala316, Lys352, Ala354, Val238, Leu242, Leu255, and Leu252, together with additional contacts involving Ser178 and Asn167. However, compared with 9e and 9i, the interaction pattern of 9m appeared less effectively organized around the key anchoring regions of the colchicine pocket, which may account for its lower potency despite maintaining a reasonable binding profile.

By contrast, compound 9l, which showed the weakest antitubulin activity among the docked comparators, adopted a completely different binding pose from the more active analogues 9e, 9i, and 9m within the colchicine binding site. Although it still exhibited some hydrophobic contacts, its markedly altered orientation prevented the ligand from reproducing the key anchoring pattern observed for the more potent compounds. This distinct binding mode likely led to suboptimal occupation of the favorable regions within the colchicine pocket and therefore provides a structural explanation for the sharp decline in activity of 9l. These findings further indicate that the combination R1 = Cl and R2 = OMe is unfavorable for productive colchicine-site recognition. The interaction diagrams of compounds 9e, 9i, 9m, and 9l are provided in the Supplementary Materials.

#### 3.3.2. ADMET prediction

**Fig. 5:**
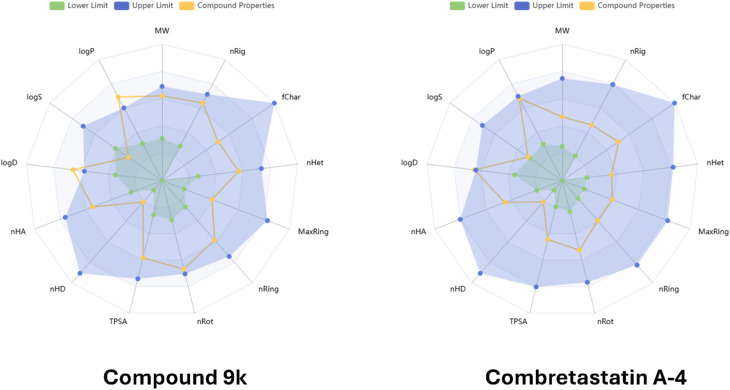
Bioavailability radar plot for compound 9k in comparison with combretastatin A-4.

*In silico* ADMET profiling of compound 9k was performed using ADMETlab 3 (ref. 48) and was further compared with the reference compound combretastatin A-4 (CA-4) to better contextualize its developability as a colchicine-site tubulin inhibitor. The predicted physicochemical descriptors showed that 9k is larger and structurally more complex than CA-4, with higher molecular weight, polarity, and flexibility (MW = 508.07 *vs.* 316.13; TPSA = 94.07 *vs.* 57.15 Å^2^; rotatable bonds = 10 *vs.* 6), together with lower predicted aqueous solubility (log *S* = −5.84 *vs.* −3.83). As shown in the bioavailability radar plots in Fig. 5, compound 9k maintains a generally acceptable bioavailability-related physicochemical profile, with differences from CA-4 mainly reflecting its distinct structural framework. These features suggest that while CA-4 represents a more compact scaffold, compound 9k still resides within a reasonable drug-like physicochemical space.

Despite these structural differences, the predicted membrane permeation descriptors of 9k remained broadly comparable to those of CA-4. Compound 9k showed predicted Caco-2 and MDCK permeabilities of −5.269 and −4.674, respectively, compared with −5.174 and −4.817 for CA-4, while PAMPA prediction was slightly higher for 9k (0.06) than for CA-4 (0.016). These results indicate that although 9k differs from CA-4 in size and polarity, its predicted cellular permeability remains within a comparable borderline-to-moderate range.

A particularly relevant difference emerged in the predicted transporter interaction profile. Compound 9k was predicted to be a non-substrate of P-glycoprotein (P-gp) and to exhibit a relatively high probability of P-gp inhibition (0.844), whereas CA-4 also behaved as a likely non-substrate but showed a lower probability of P-gp inhibition (0.593). Moreover, 9k was predicted to inhibit MRP1 but not BCRP, while CA-4 showed inhibition of both MRP1 and BCRP. This suggests that the transporter profile of 9k may be advantageous in the context of multidrug resistance, since the combination of predicted P-gp non-substrate behavior and stronger P-gp inhibitory potential may help maintain intracellular drug exposure in resistant tumor cells.

The predicted distribution profile also distinguished the two compounds. Compound 9k showed very high plasma protein binding (PPB = 98.768%) and a very low fraction unbound (Fu = 1.082%), whereas CA-4 displayed lower PPB (74.467%) and a substantially higher Fu (19.266%). In addition, 9k had a very low predicted probability of blood–brain barrier penetration (BBB = 0.007) compared with CA-4 (0.457), indicating that 9k may have more restricted central nervous system distribution. Therefore, relative to CA-4, 9k may circulate predominantly in a protein-bound form and display lower free systemic exposure.

The metabolic liability profile of 9k also differed from that of CA-4. Compound 9k was predicted to inhibit multiple CYP isoforms, including CYP1A2, CYP2C19, CYP2C9, CYP2B6, CYP2C8, and CYP3A4, and was also predicted to behave as a CYP2C9 substrate. In contrast, CA-4 showed a narrower CYP inhibition profile, mainly involving CYP1A2, CYP2B6, and CYP2C8, although it was predicted to act as a substrate for CYP2C9, CYP2D6, and CYP3A4. Furthermore, 9k showed a high probability of human liver microsomal instability (HLM stability = 0.841), suggesting possible metabolic vulnerability. These findings indicate that 9k may carry broader metabolism-related liabilities than CA-4 and would likely benefit from future structural optimization.

With respect to excretion, 9k showed lower predicted plasma clearance than CA-4 (2.29 *vs.* 8.147 mL min^−1^ kg^−1^), while both compounds exhibited a short predicted half-life (1.156 *vs.* 1.452 h). Lower clearance may partially compensate for the high protein binding of 9k, although its overall exposure profile would still require experimental confirmation.

The toxicity predictions further suggested that 9k may have more pronounced liabilities than CA-4 in several categories. In particular, 9k showed markedly higher predicted risk for drug-induced liver injury (0.999 *vs.* 0.109), genotoxicity (0.999 *vs.* 0.083), carcinogenicity (0.815 *vs.* 0.468), and drug-induced nephrotoxicity (0.852 *vs.* 0.702). On the other hand, some endpoints were less unfavorable for 9k, such as eye irritation and respiratory toxicity. Overall, these results indicate that 9k does not exhibit a superior virtual ADMET profile relative to CA-4. Nevertheless, its distinct transporter-related behavior, especially its predicted P-gp non-substrate character combined with stronger P-gp inhibitory potential, still supports its value as a mechanistically interesting lead scaffold for further optimization rather than as a direct ADMET-improved analogue of CA-4.

### 3.4. Structure activity relationship (SAR) analysis

The structure–activity relationship (SAR) analysis revealed the following trends:
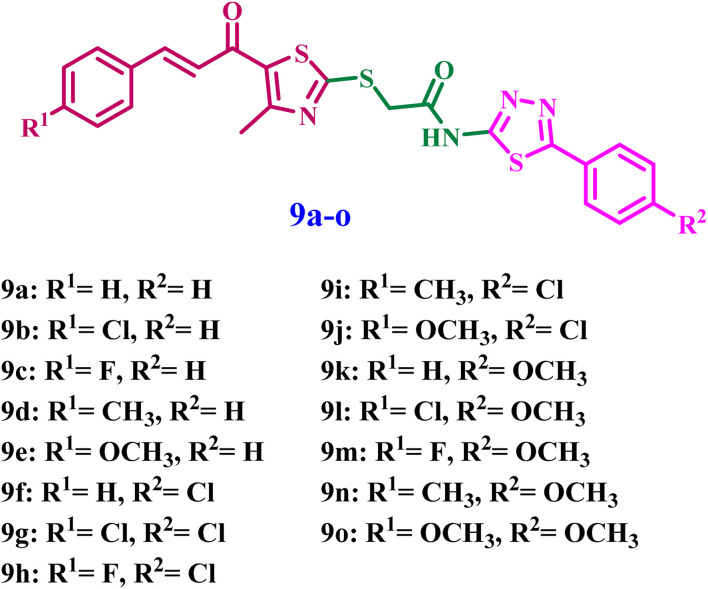


• Substitution at both R^1^ and R^2^ strongly affected activity. The most active analogue was 9k (R^1^ = H, R^2^ = OMe), indicating that this substitution pattern provides the best overall fit for the series.

• In the R^2^ = H series, the activity order was 9e (OMe) > 9b (Cl) > 9d (Me) > 9c (F) > 9a (H), showing that R^1^ = OMe is the most favorable substituent in this subgroup. This agrees with docking study, where 9e showed a productive pose supported by a good balance of polar anchoring and hydrophobic stabilization.

• In the R^2^ = Cl series, the order became 9i (Me) > 9g (Cl) > 9h (F) > 9j (OMe) > 9f (H), indicating that R^1^ = Me is optimal when R^2^ = Cl. Docking of 9i also suggested a highly favorable binding arrangement, which is consistent with its strong tubulin inhibitory activity.

• In the R^2^ = OMe series, the trend was 9k (H) >> 9m (F) > 9n (Me) > 9o (OMe) > 9l (Cl), showing that an unsubstituted R1 is most favorable in this subgroup. Docking supported this trend, as 9m retained a reasonably favorable orientation, whereas 9l adopted a less productive pose, explaining its marked loss of activity.

Overall, the results show that the effects of R^1^ and R^2^ are interdependent rather than additive. The best activity was observed with substitution patterns that allowed a more favorable docking orientation, as seen for 9k, 9i, and 9e, whereas mismatched combinations such as 9l were detrimental despite the presence of multiple substituents.

## 4. Conclusion

This study presents thiazole-based derivatives 9a–o as a promising category of colchicine-site tubulin inhibitors that demonstrate low-micromolar antiproliferative action while exhibiting minimal toxicity to normal MCF-10A cells at 50 µM. In this series, compound 9k was identified as the most effective analogue, exhibiting significant tubulin inhibition (IC_50_ = 1.56 µM), enhanced antiproliferative activity against HeLa, HCT-116, and A-549 cancer cell lines, and the ability to activate the intrinsic apoptotic pathway *via* Bax upregulation, Bcl-2 downregulation, and substantial caspase-9/3 activation. Molecular docking into the colchicine binding site corroborated our findings by demonstrating a favorable binding conformation for 9k, featuring critical interactions typical of high-affinity CBS ligands. The complementary *in silico* ADMET analysis further validated 9k as a drug-like candidate, demonstrating good oral bioavailability and a favorable P-gp transporter profile aligned with the expected characteristics of tubulin-targeted anticancer medicines. All of these findings highlight 9k as a prospective framework for targeted cancer therapy. Future research should emphasize *in vivo* efficacy investigations and structural improvements to augment its potency and selectivity against resistant tumor models.

## Conflicts of interest

The authors declare no competing interests.

## Supplementary Material

RA-016-D6RA01355D-s001

## Data Availability

The authors assert that any data supporting this study can be found in the supplementary information (SI). Supplementary information is available. See DOI: https://doi.org/10.1039/d6ra01355d.
