# Supplementary material for: Design, synthesis, and antiproliferative activity of novel thiazole-based derivatives as tubulin polymerization inhibitors targeting the colchicine binding site
Source: RSC Adv. 2026 May 12;16(27):25103–15. doi: 10.1039/d6ra01355d (PMC13165019; doi:10.1039/d6ra01355d)
Supplement: RA-016-D6RA01355D-s001 [file RA-016-D6RA01355D-s001.pdf]

Supplementary Information (SI) for RSC Advances.
This journal is © The Royal Society of Chemistry 2026

Design, synthesis, and antiproliferative activity of novel thiazole-based derivatives as tubulin polymerization inhibitors targeting the colchicine binding site

Lamya H. Al-Wahaibi[1], Ali M. Elshamsy[2], Taha F.S. Ali[3], Bahaa G. M. Youssif*[4], Stefan Bräse[5]*, Mohamed Abdel-Aziz*[3,6], Nawal A. El-Koussi[7]

[1]Department of Chemistry, College of Sciences, Princess Nourah bint Abdulrahman University, Riyadh 11671, Saudi Arabia; [2]Pharmceutical Chemistry Department, Faculty of Pharmacy, Deraya University, Minia, Egypt; [3]Medicinal Chemistry Department, Faculty of Pharmacy, Minia University, Minia 61519, Egypt; [4]Department of Pharmaceutical Organic Chemistry, Faculty of Pharmacy, Assiut University, Assiut-71526, Egypt; [5]Institute of Biological and Chemical Systems, IBCS-FMS, Karlsruhe Institute of Technology, 76131 Karlsruhe, Germany; [6]Medicinal Chemistry Department, Faculty of Pharmacy, International Minia University, Minia, Egypt; [7]Department of Pharmaceutical Medicinal Chemistry, Faculty of Pharmacy, Assiut University, Assiut, Egypt.

*To whom correspondence should be addressed:

Mohamed Abdel-Aziz, Ph.D. Department of Medicinal Chemistry, Faculty of Pharmacy, Minia University, 61519-Minia, Egypt.

Tel.: +2101003311327; E-mail address: abulnil@hotmail.com

Bahaa G. M. Youssif, Ph.D. Pharmaceutical Organic Chemistry Department, Faculty of Pharmacy, Assiut University, Assiut 71526, Egypt.

Tel.: +201098294419; E-mail address: bgyoussif2@gmail.com

Stefan Bräse

Institute of Biological and Chemical Systems, IBCS-FMS, Karlsruhe Institute of Technology, 76131 Karlsruhe, Germany. E-mail: braese@kit.edu

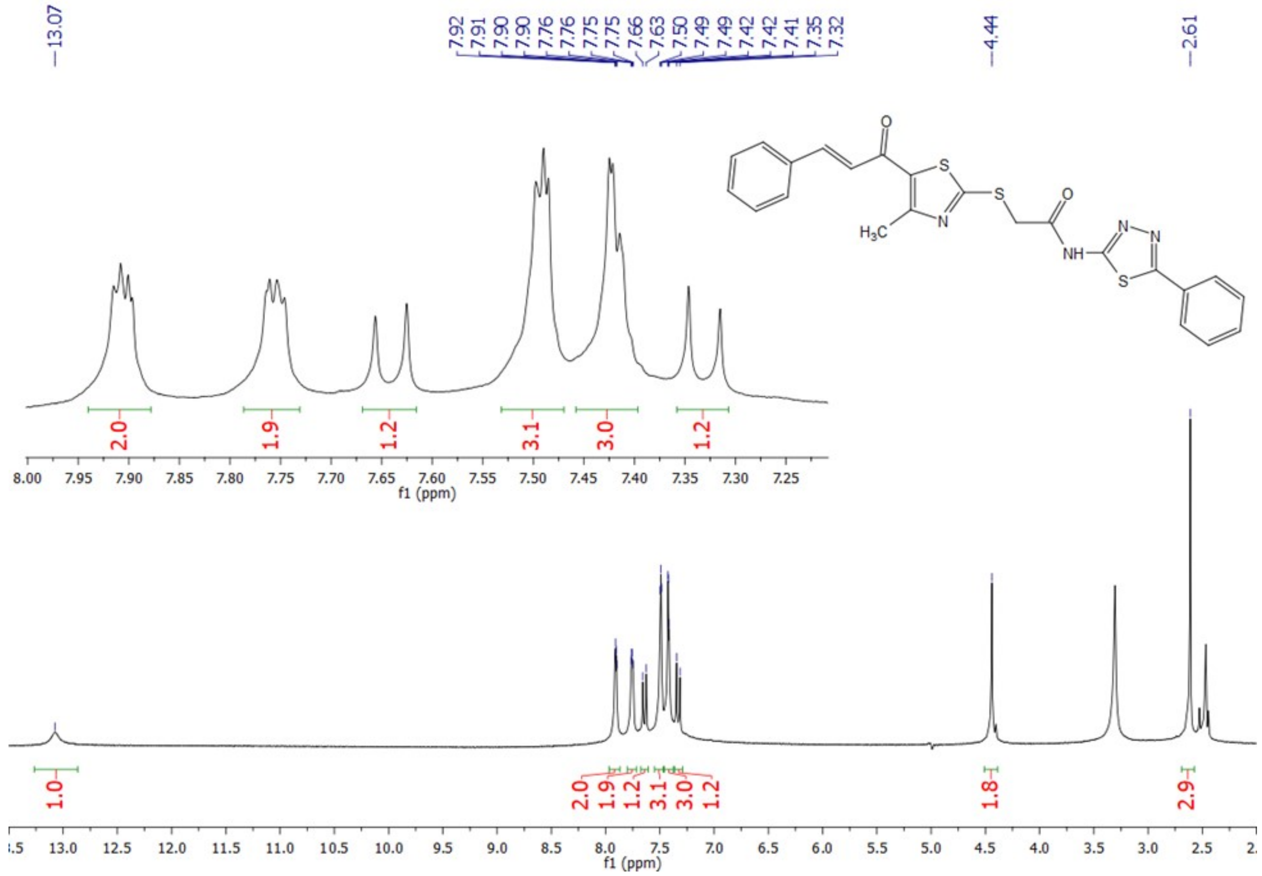

Figure S1. ^{1}H NMR spectrum of compound **9a**

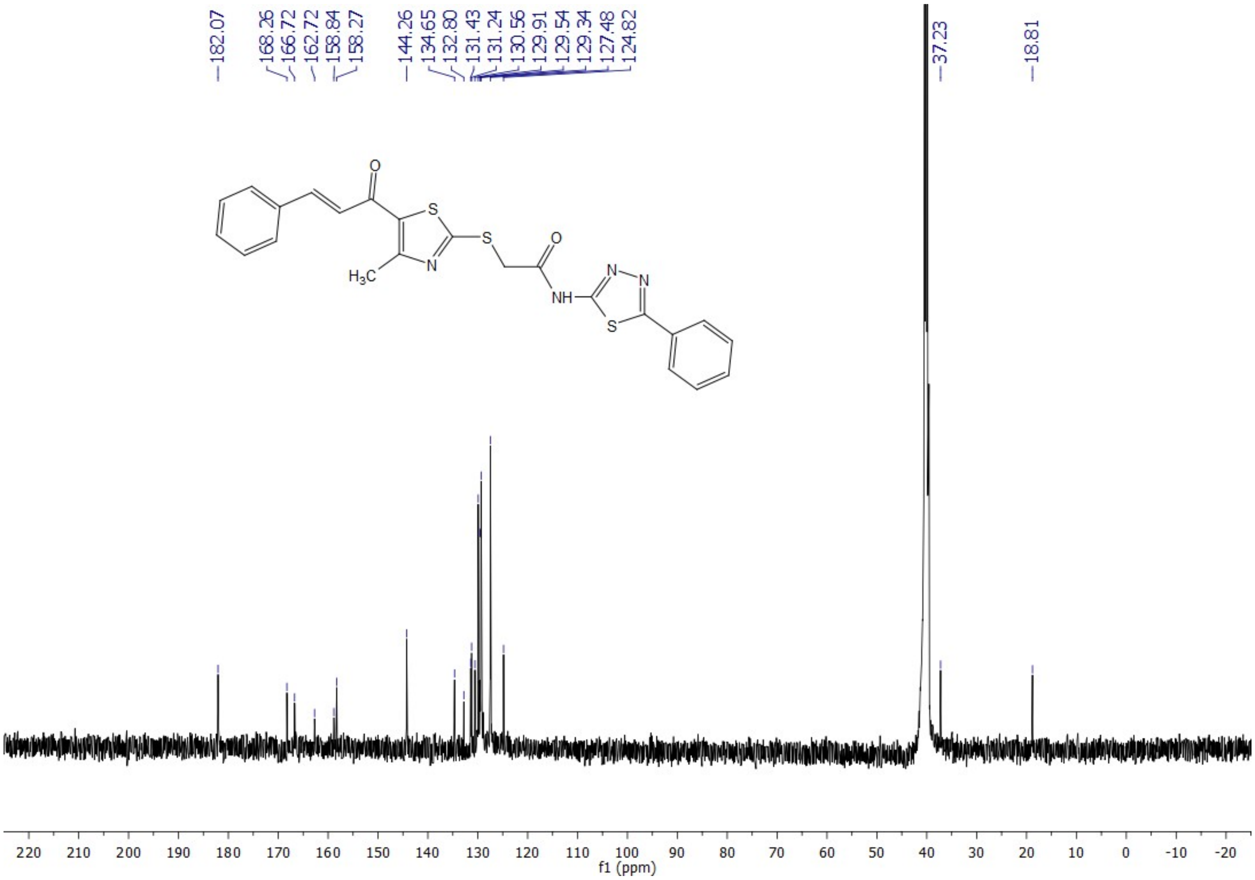

Figure S2. ^{13}C NMR spectrum of compound **9a**

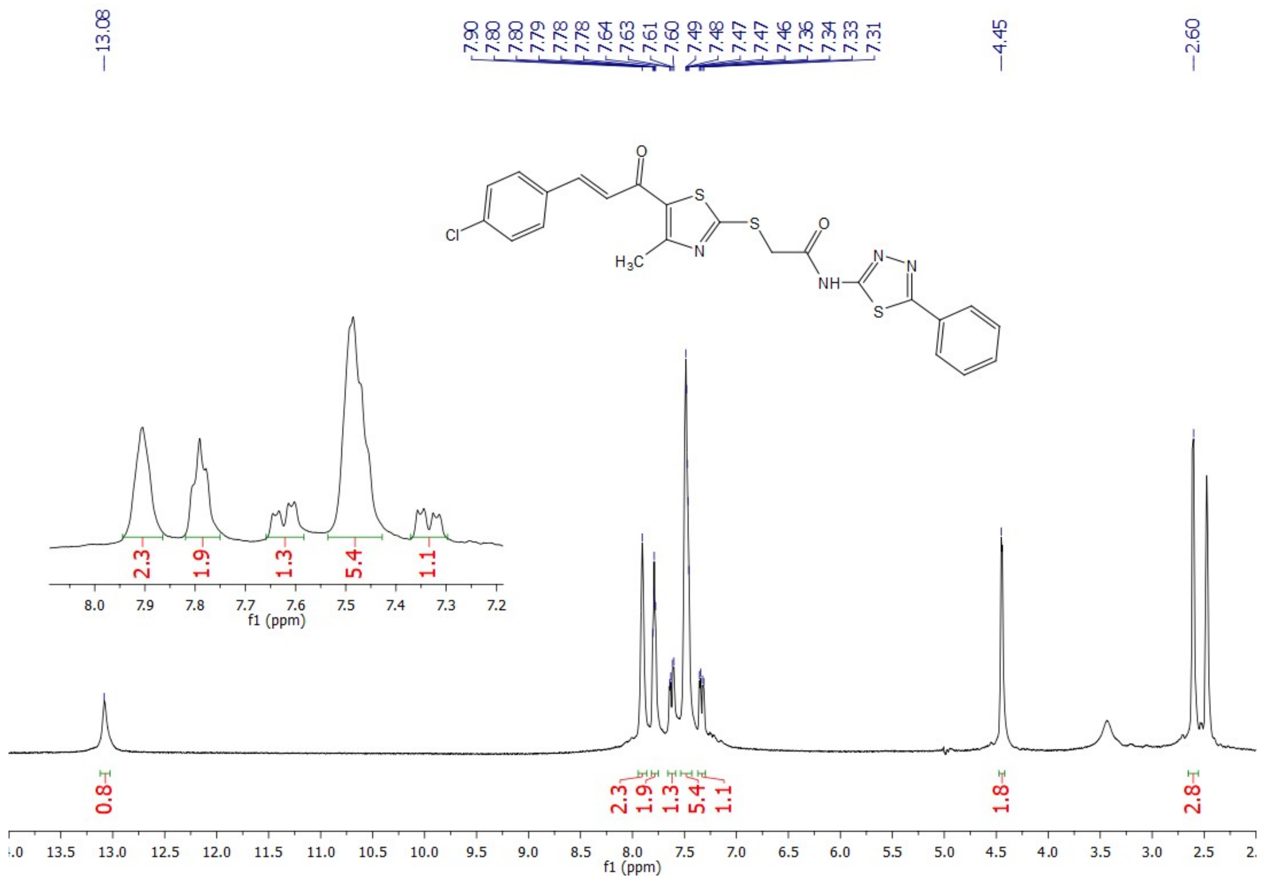

Figure S3. ^{1}H NMR spectrum of compound **9b**

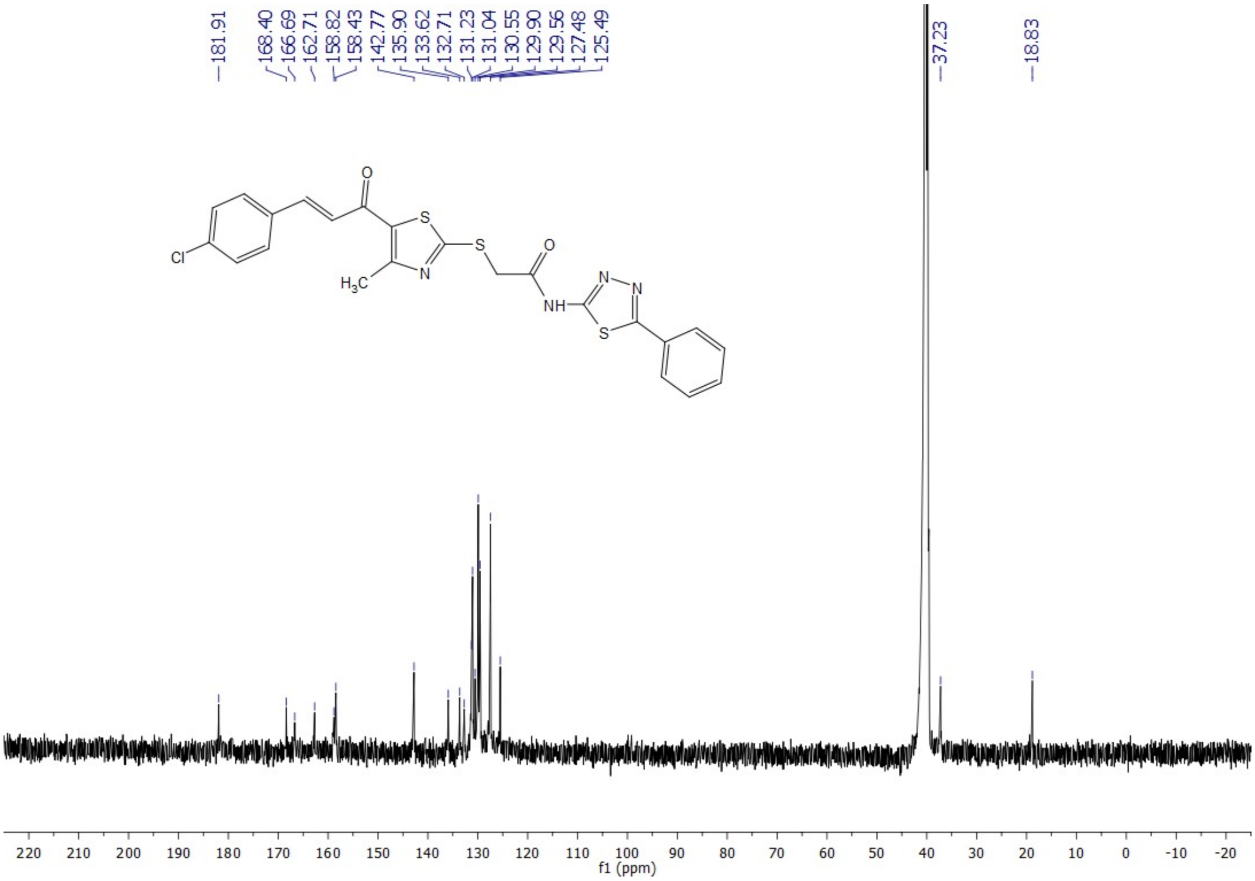

Figure S4. ^{13}C NMR spectrum of compound **9b**

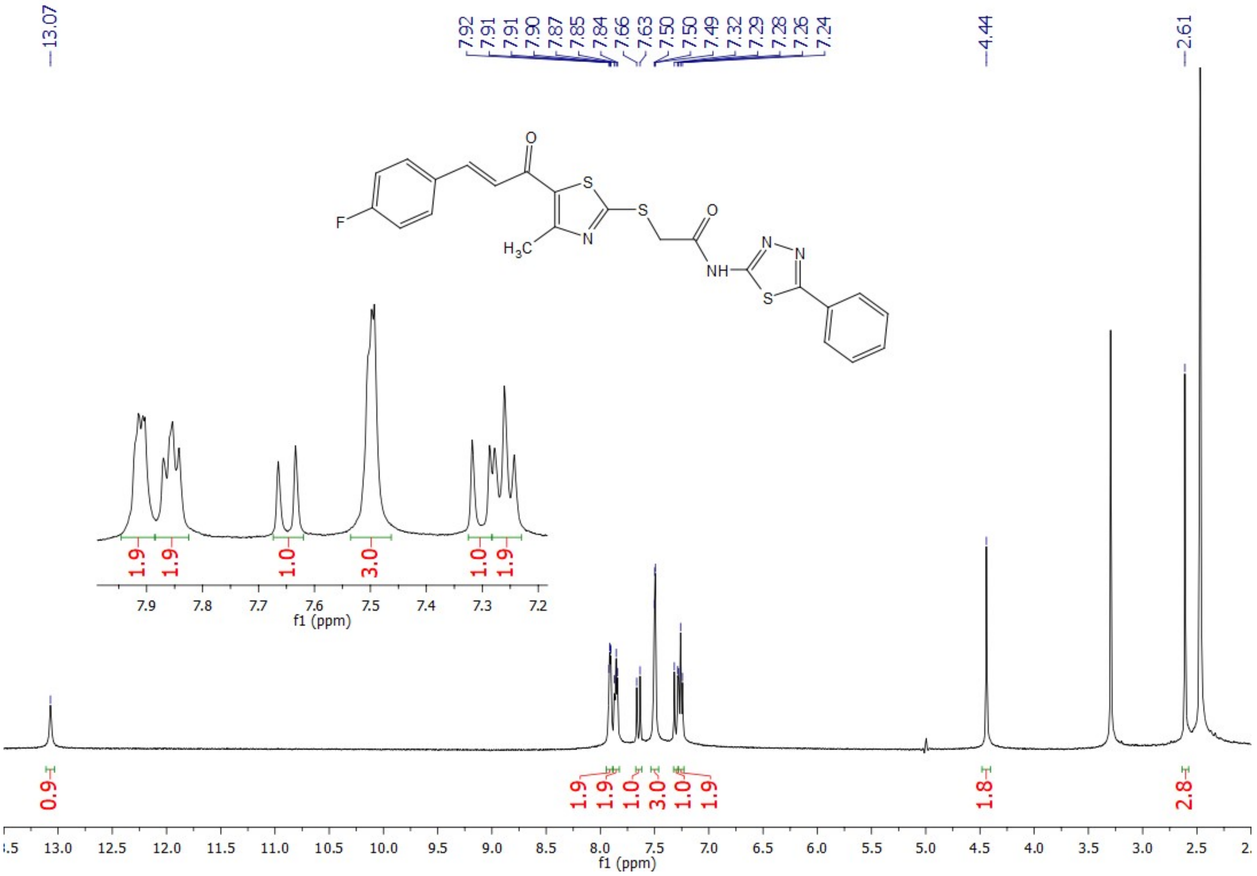

Figure S5. ^{1}H NMR spectrum of compound **9c**

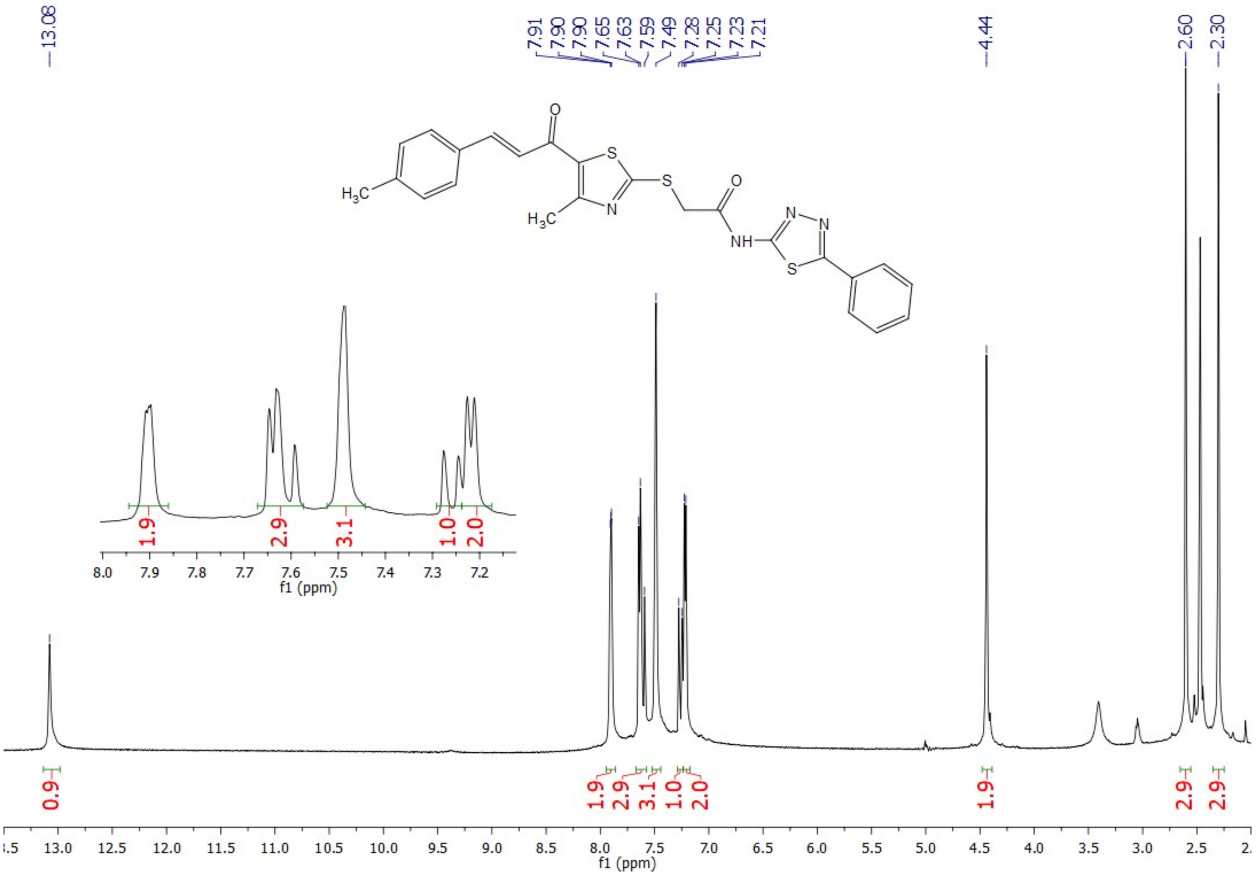

Figure S6. ^{1}H NMR spectrum of compound **9d**

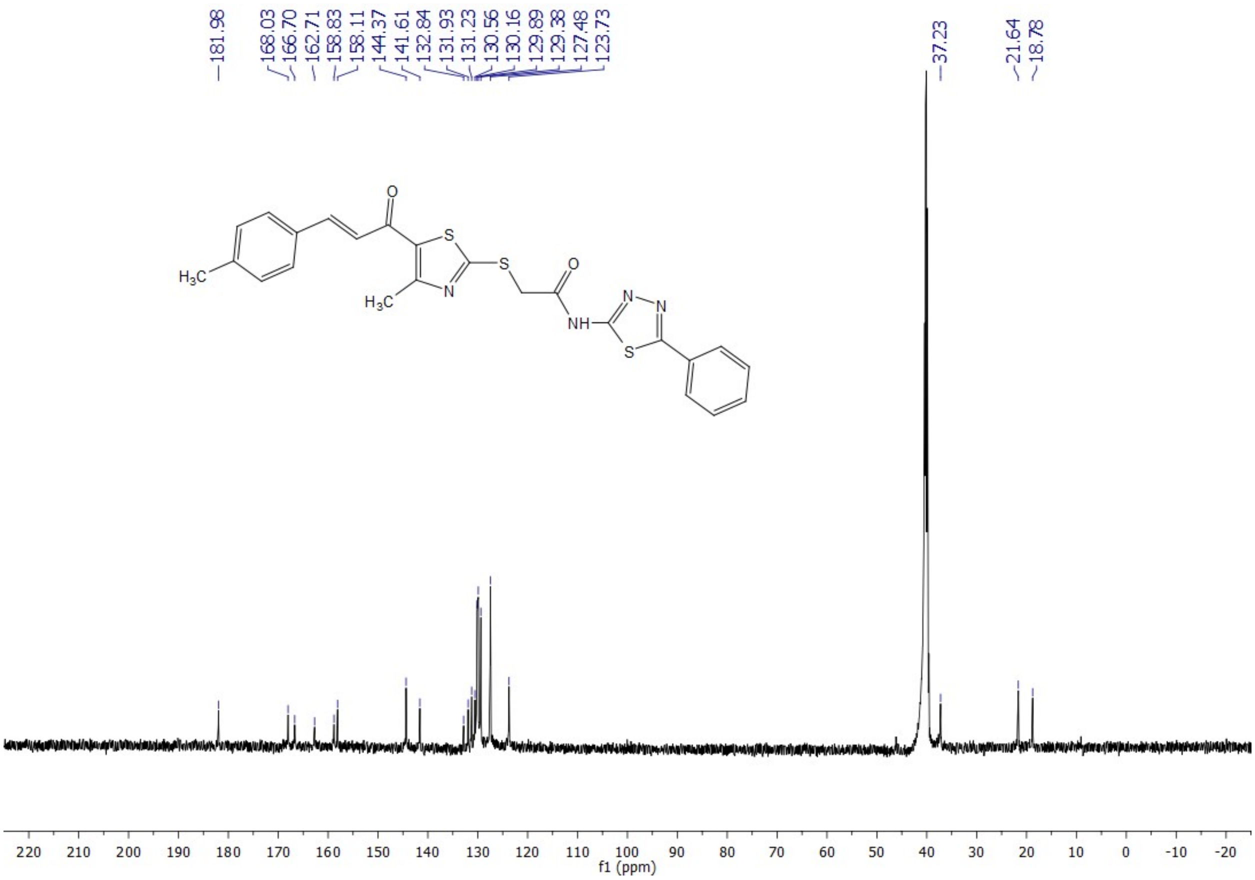

Figure S7. ^{13}C NMR spectrum of compound **9d**

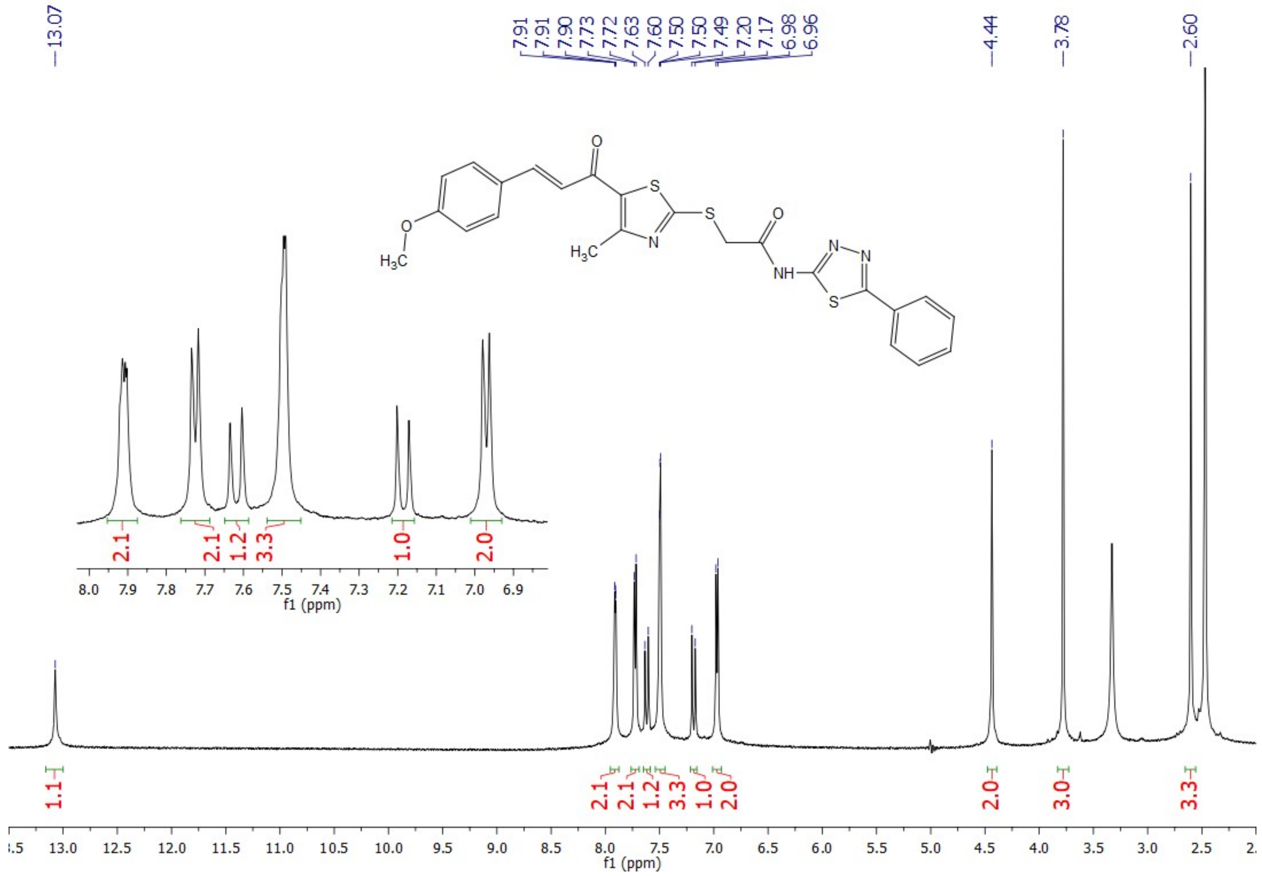

Figure S8. ^{1}H NMR spectrum of compound **9e**

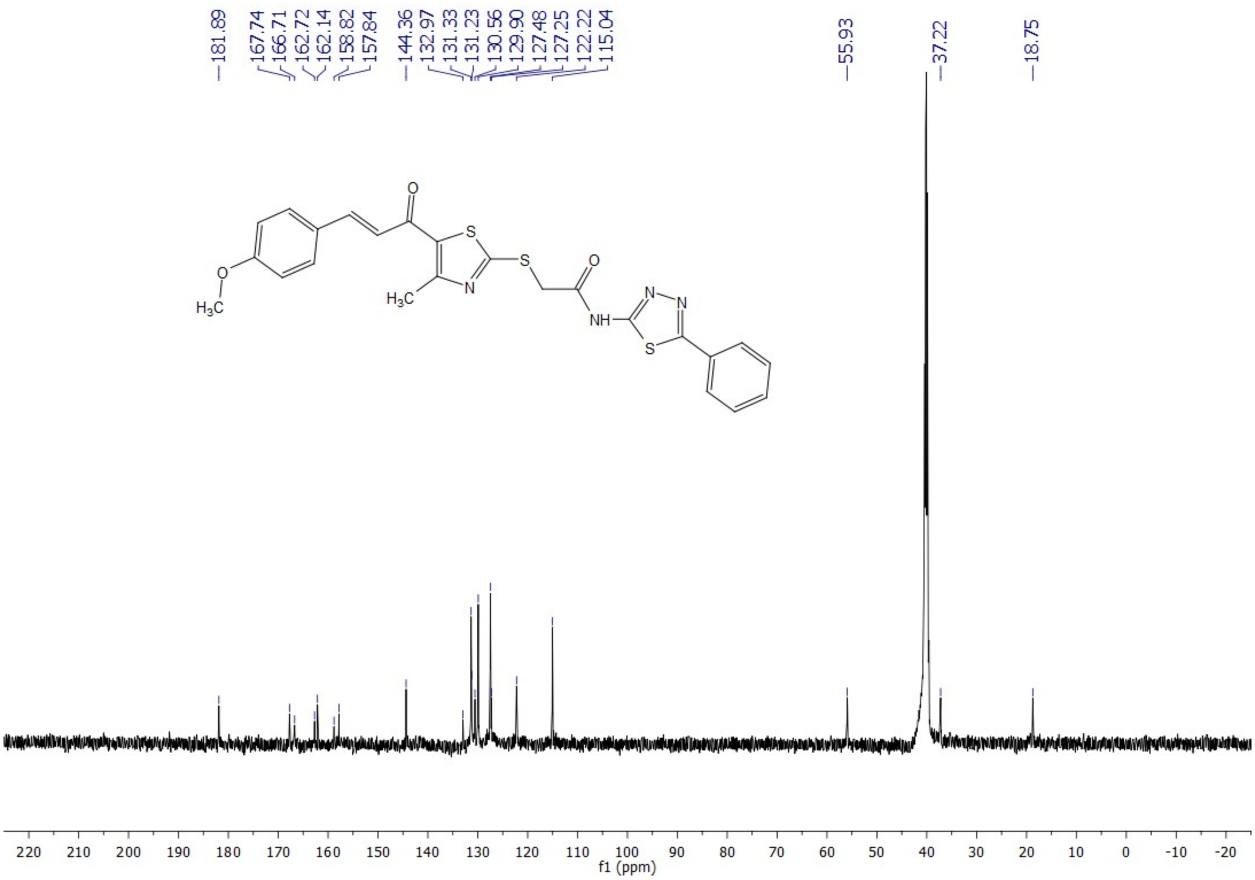

Figure S9. ^{13}C NMR spectrum of compound **9e**

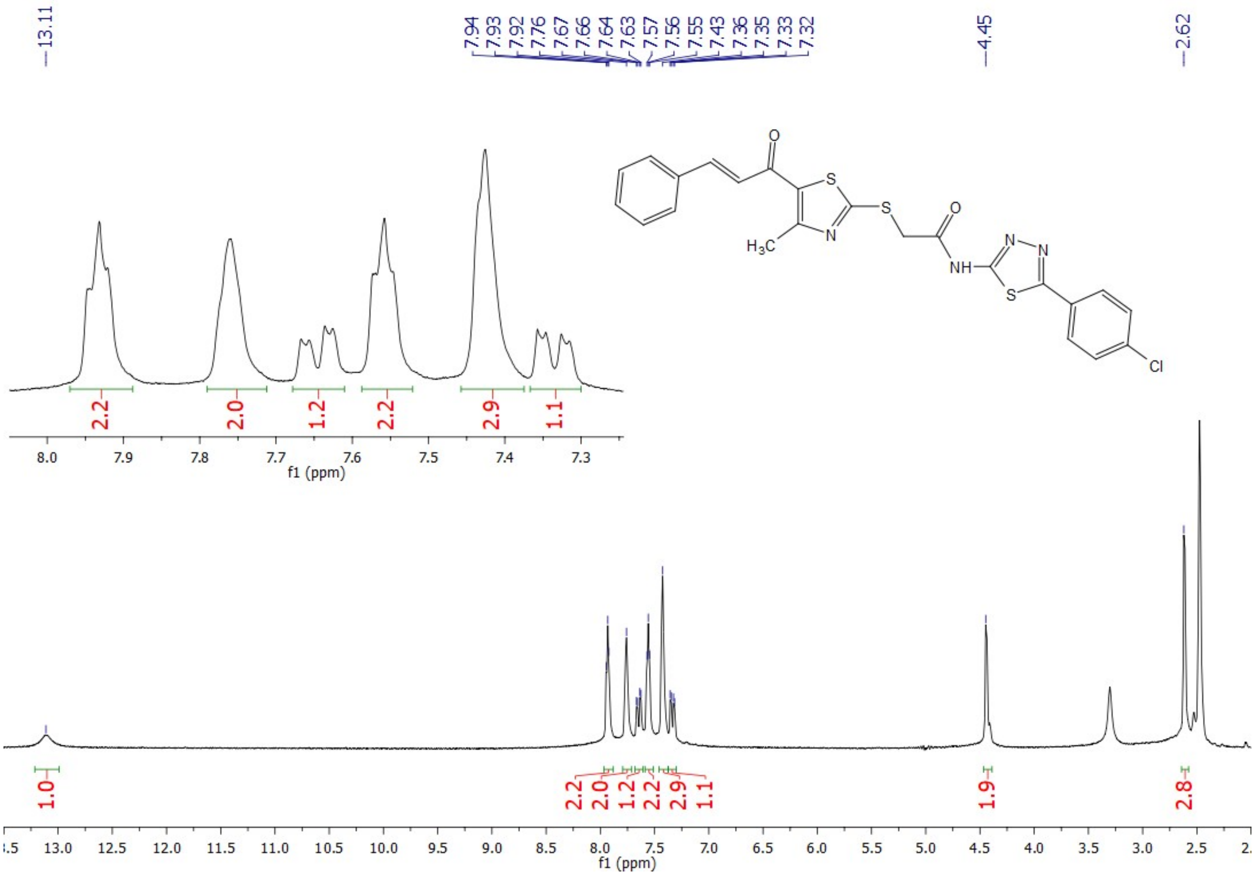

Figure S10. ^{1}H NMR spectrum of compound **9f**

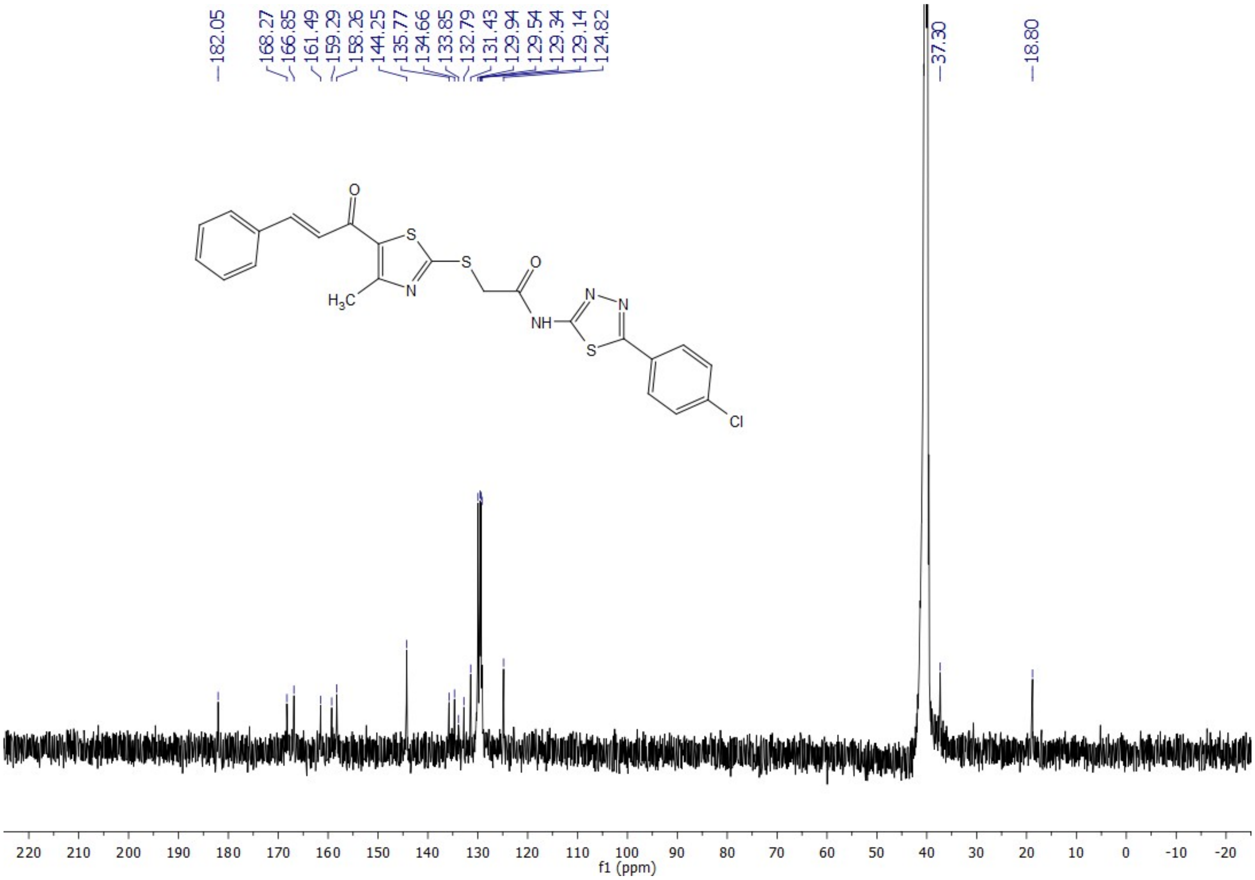

Figure S11. ^{13}C NMR spectrum of compound **9f**

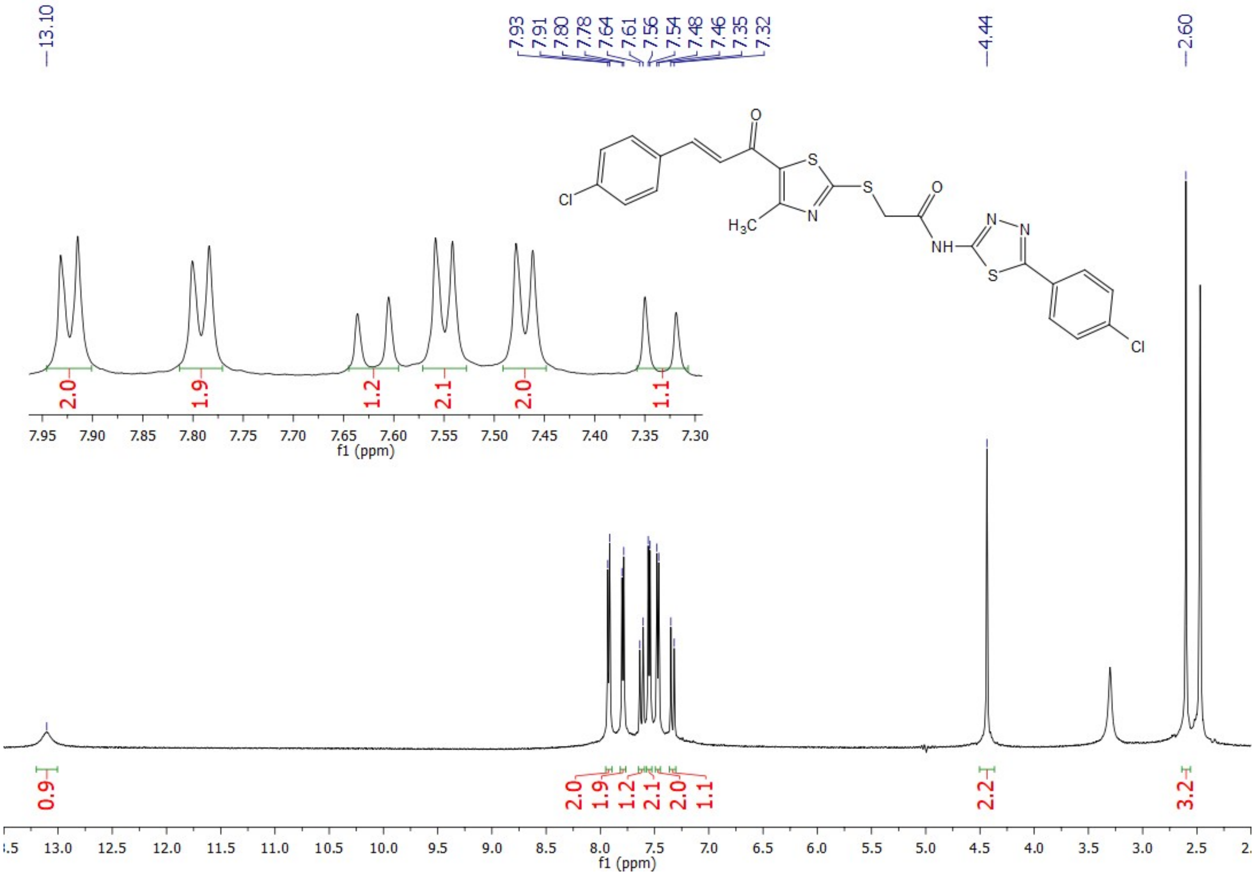

Figure S12. ^{1}H NMR spectrum of compound **9g**

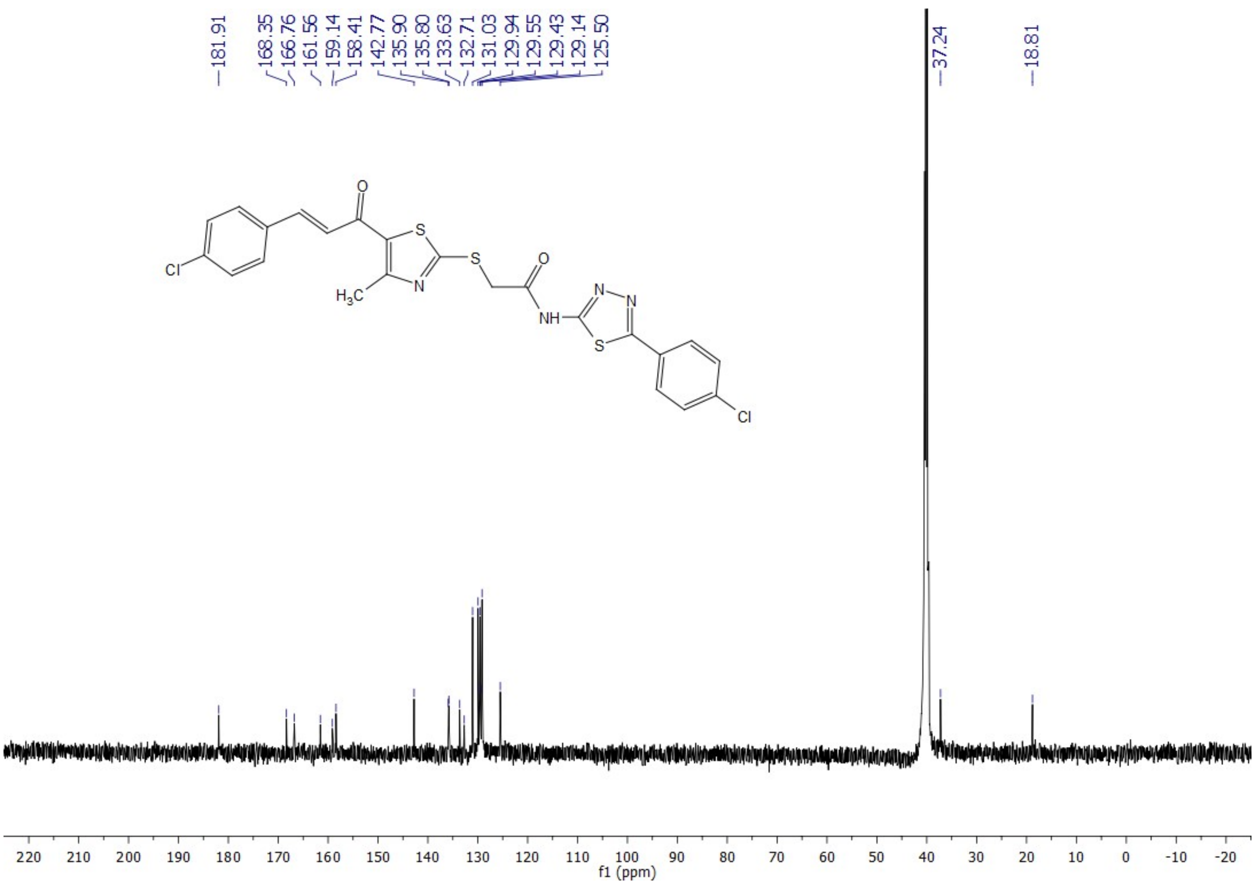

Figure S13. ^{13}C NMR spectrum of compound **9g**

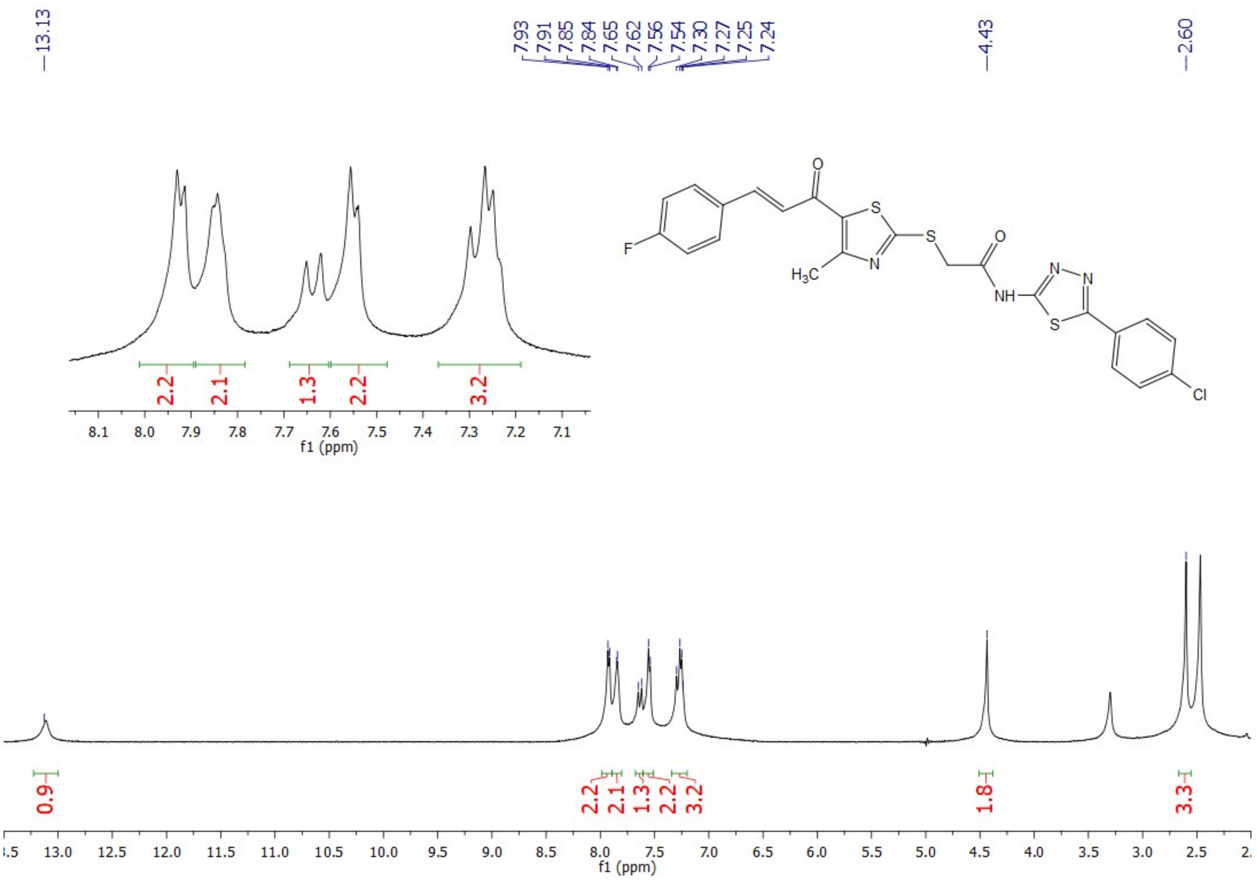

Figure S14. ^{1}H NMR spectrum of compound **9h**

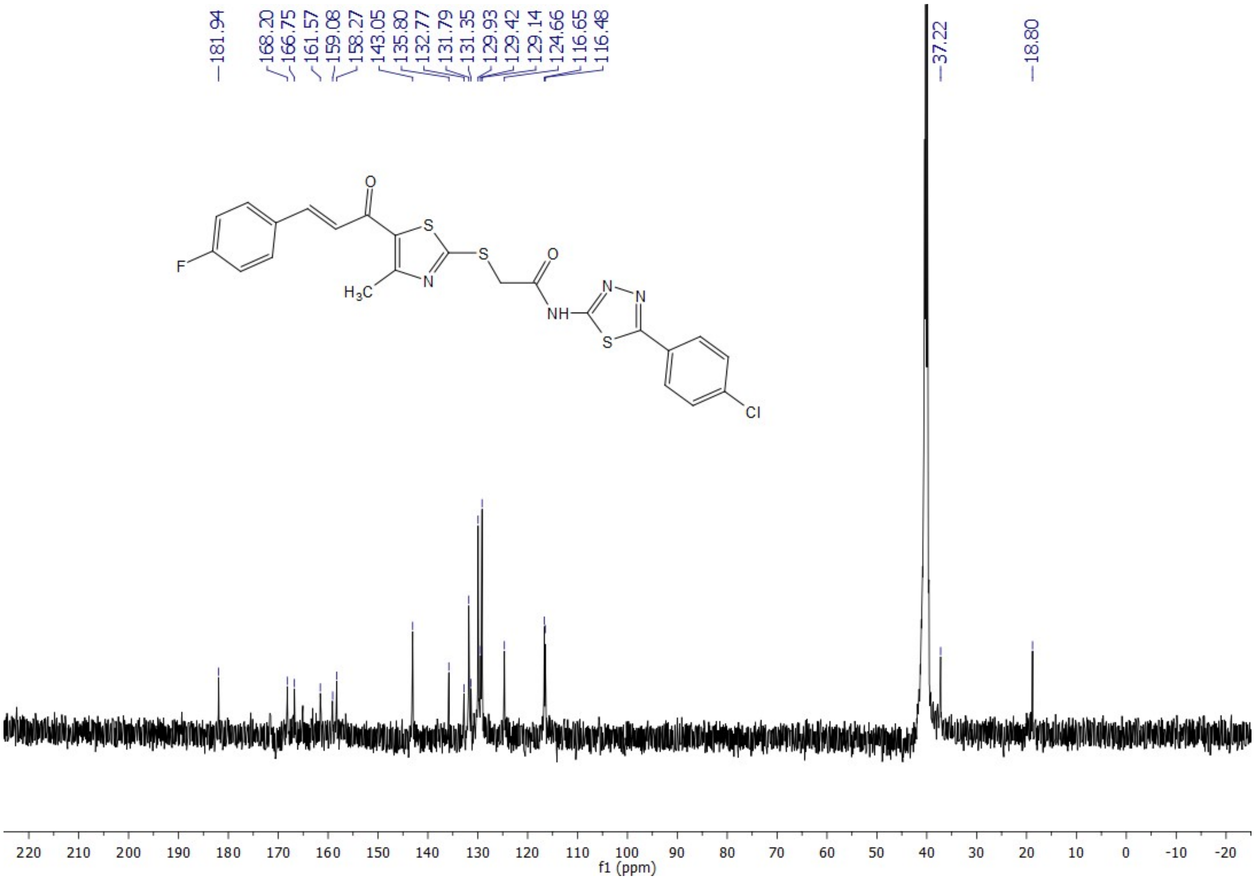

Figure S15. ^{13}C NMR spectrum of compound **9h**

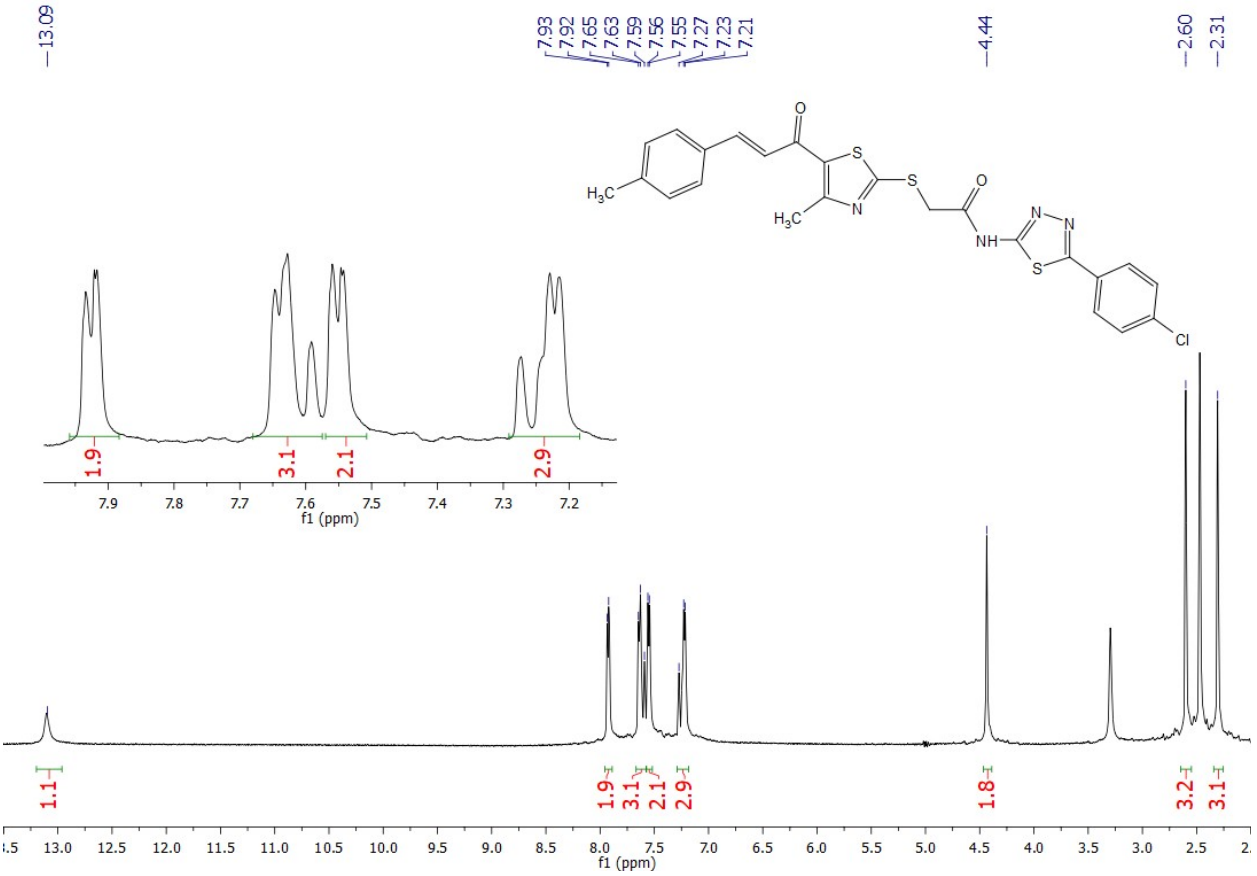

Figure S16. ^{1}H NMR spectrum of compound **9i**

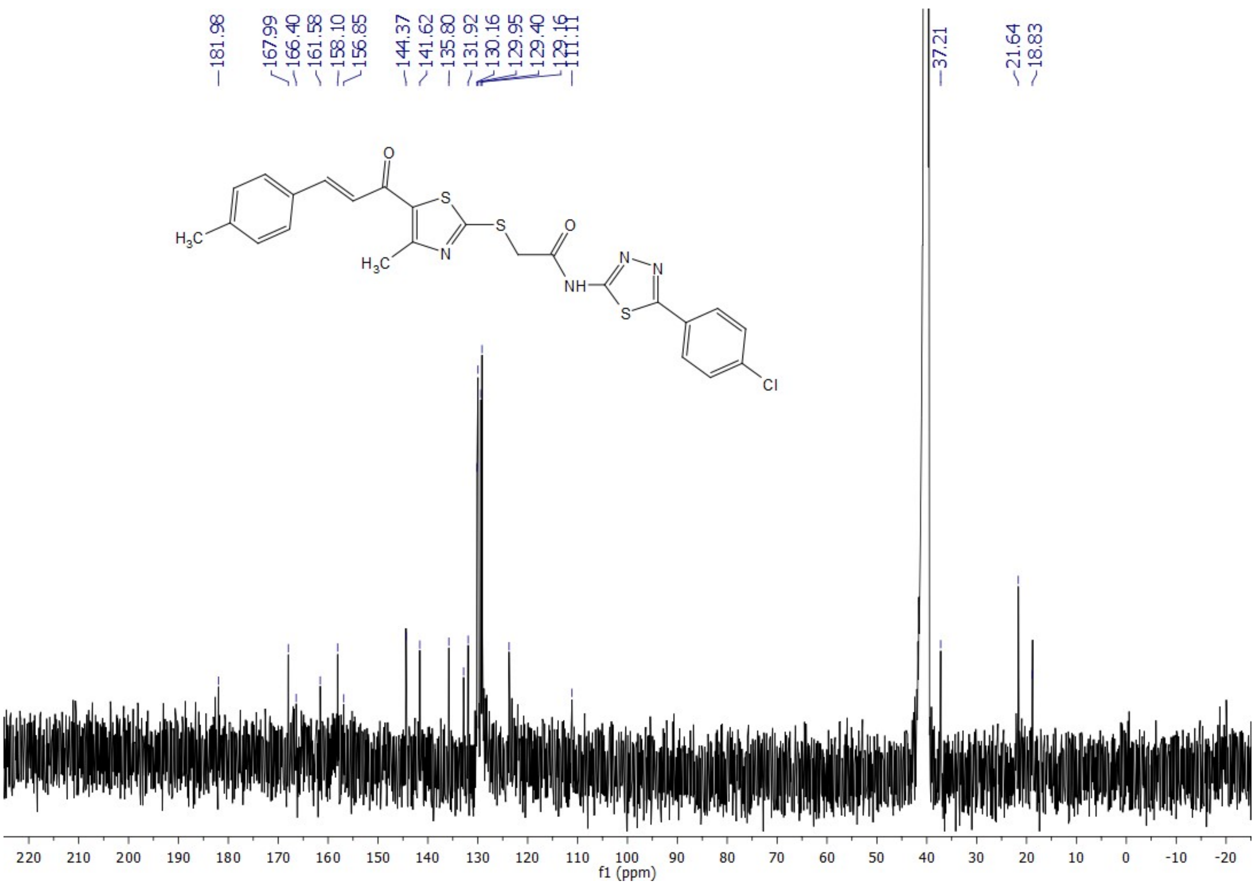

Figure S17. ^{13}C NMR spectrum of compound **9i**

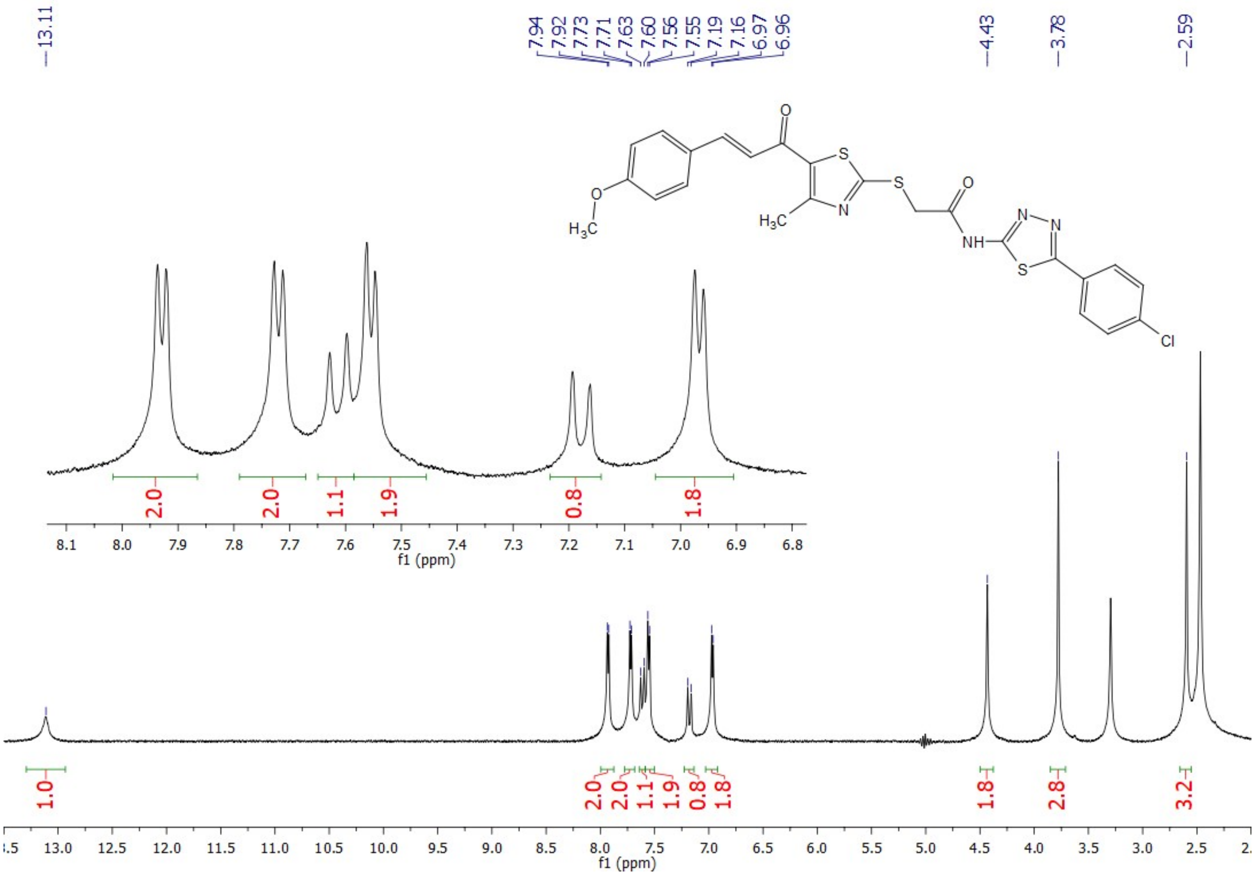

Figure S18. ^{1}H NMR spectrum of compound **9j**

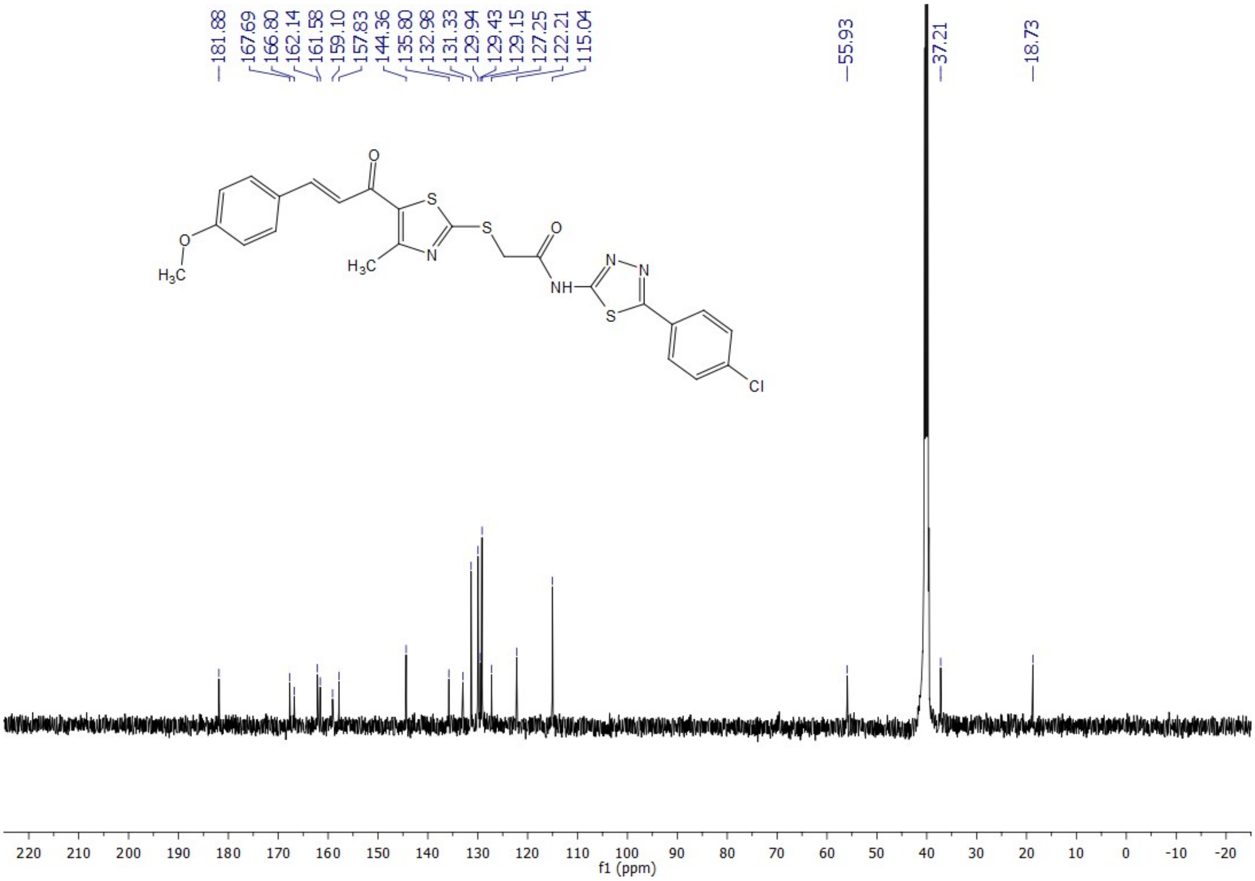

Figure S19. ^{13}C NMR spectrum of compound **9j**

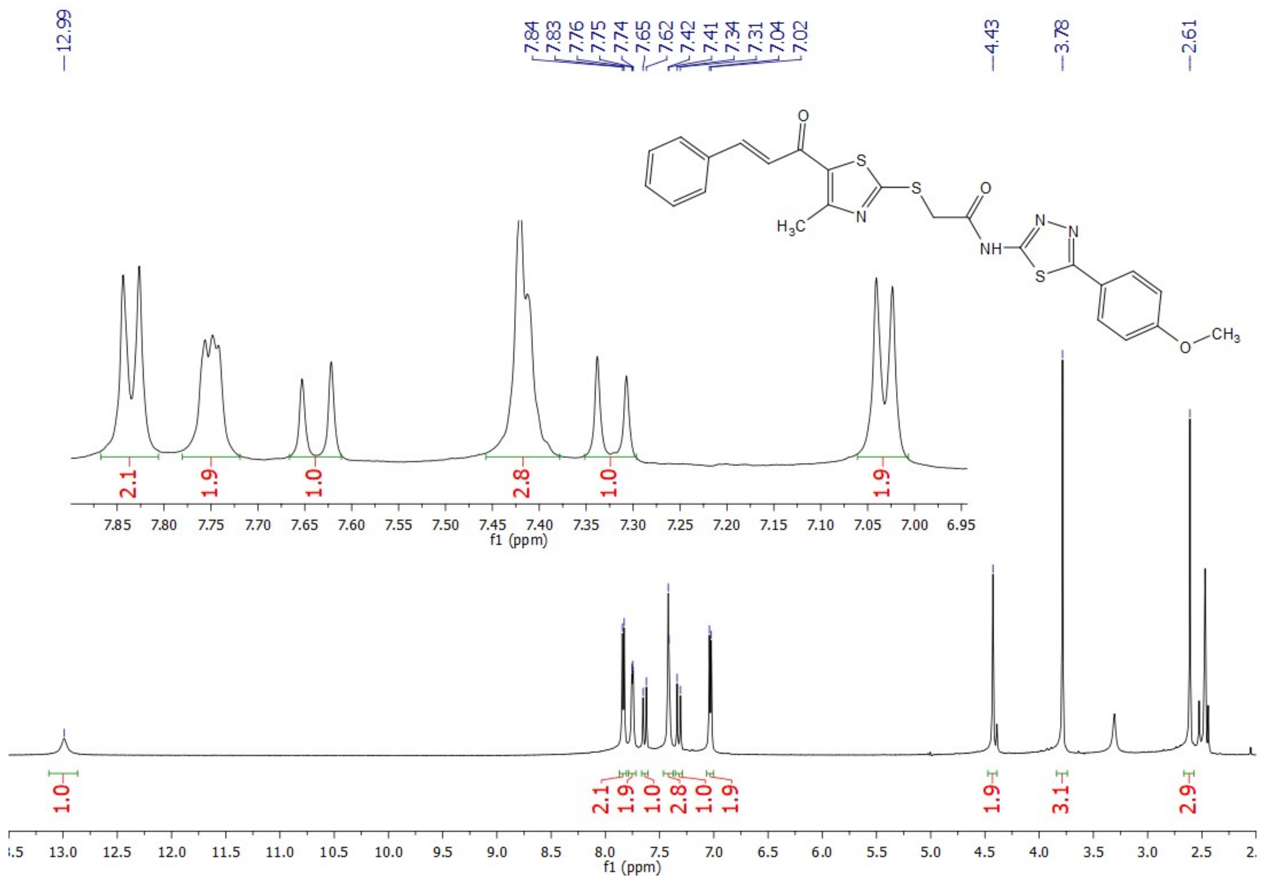

Figure S20. ^{1}H NMR spectrum of compound **9k**

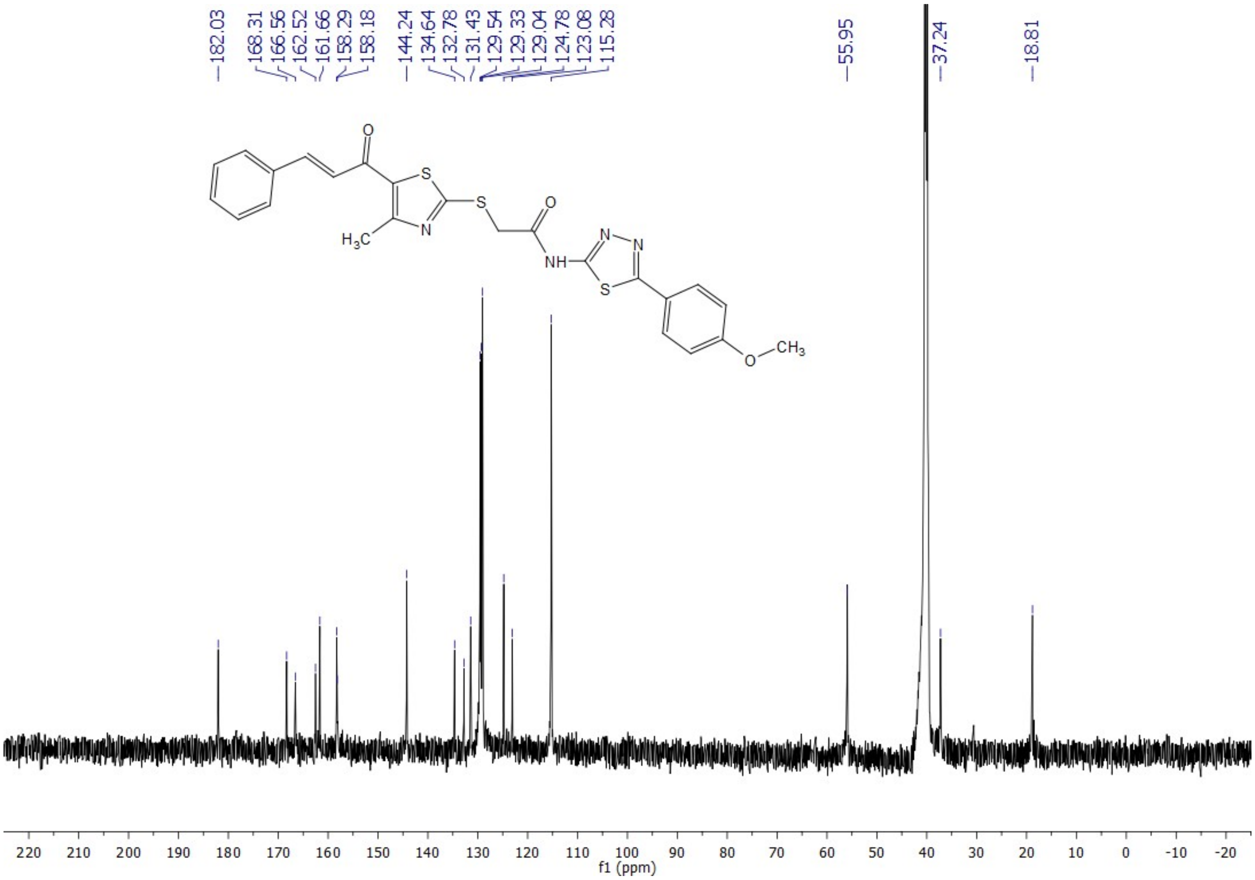

Figure S21. ^{13}C NMR spectrum of compound **9k**

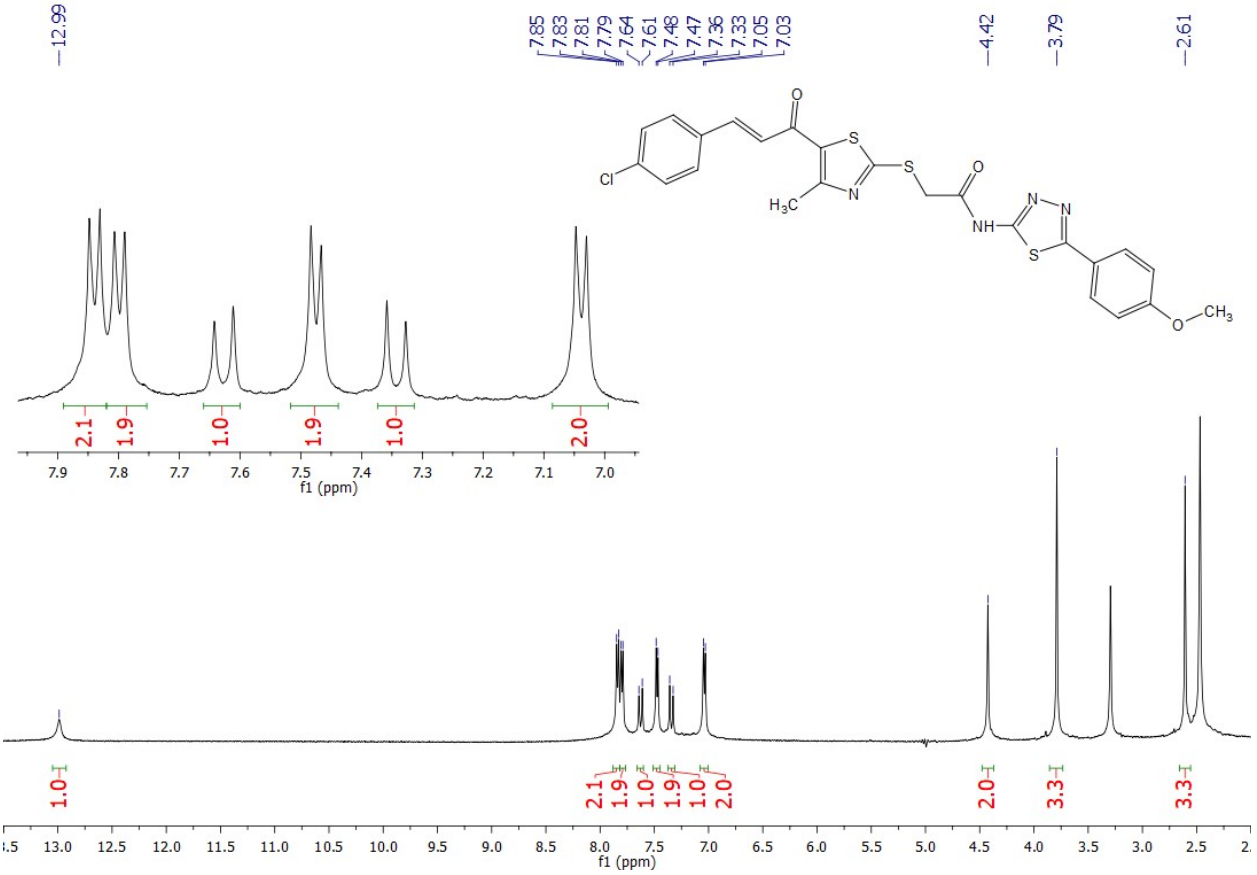

Figure S22. ^{1}H NMR spectrum of compound **9l**

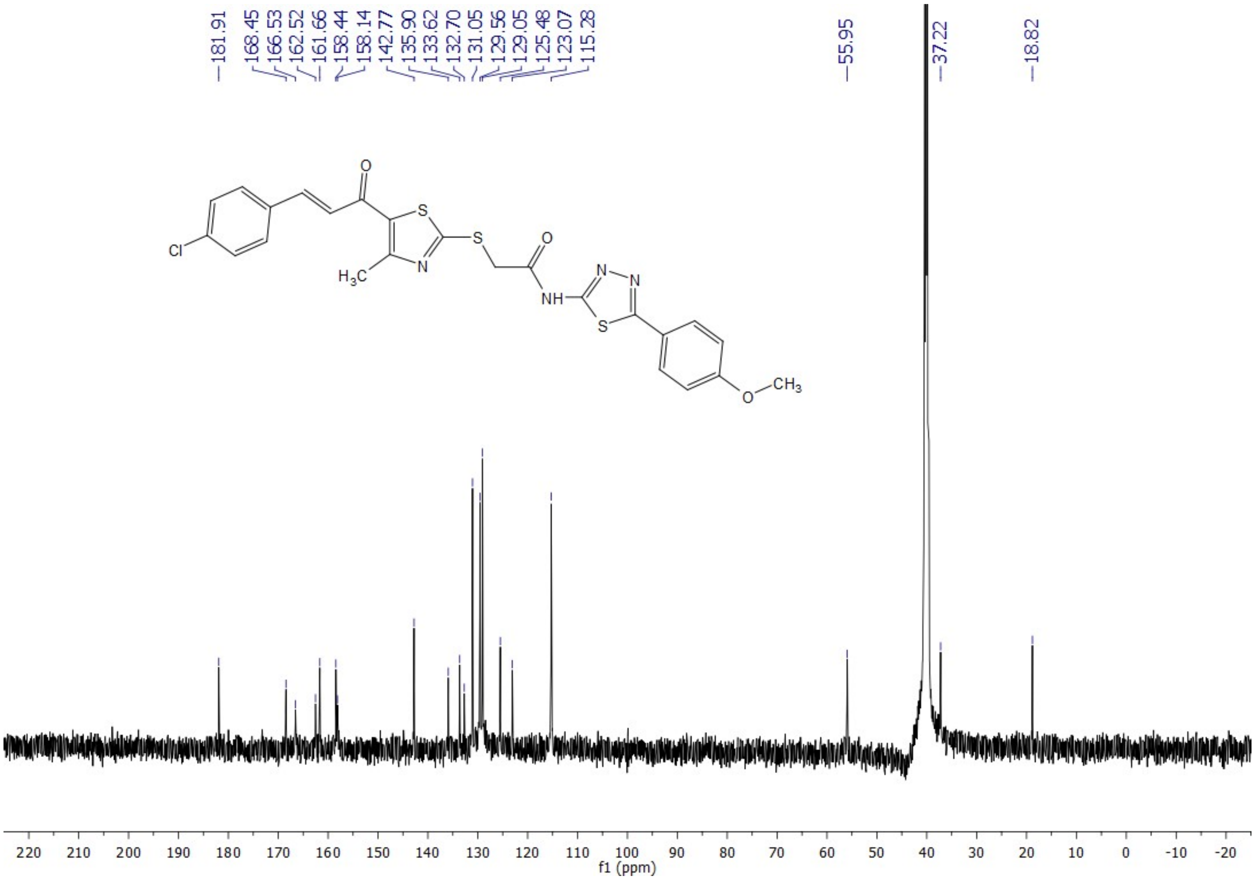

Figure S23. ^{13}C NMR spectrum of compound **9l**

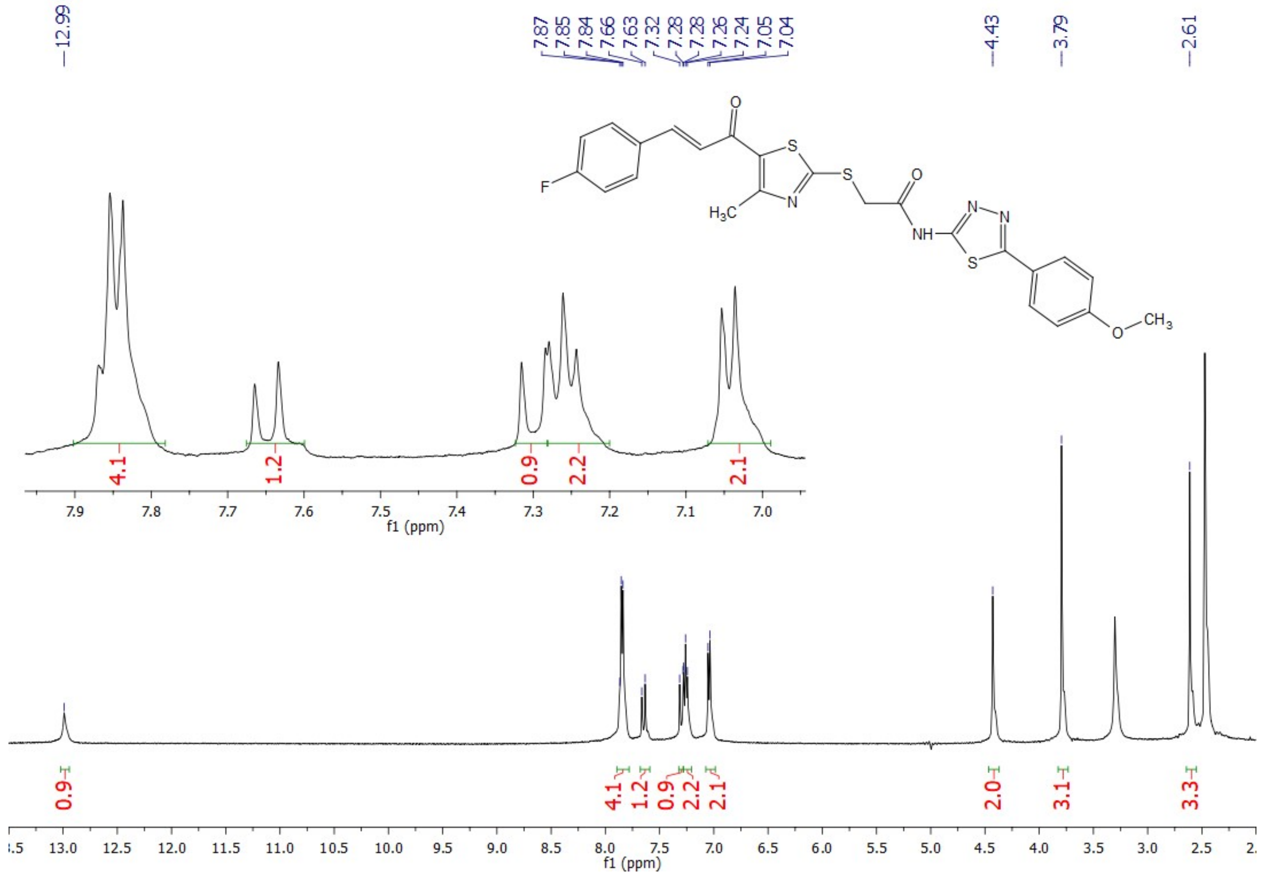

Figure S24. ^{1}H NMR spectrum of compound **9m**

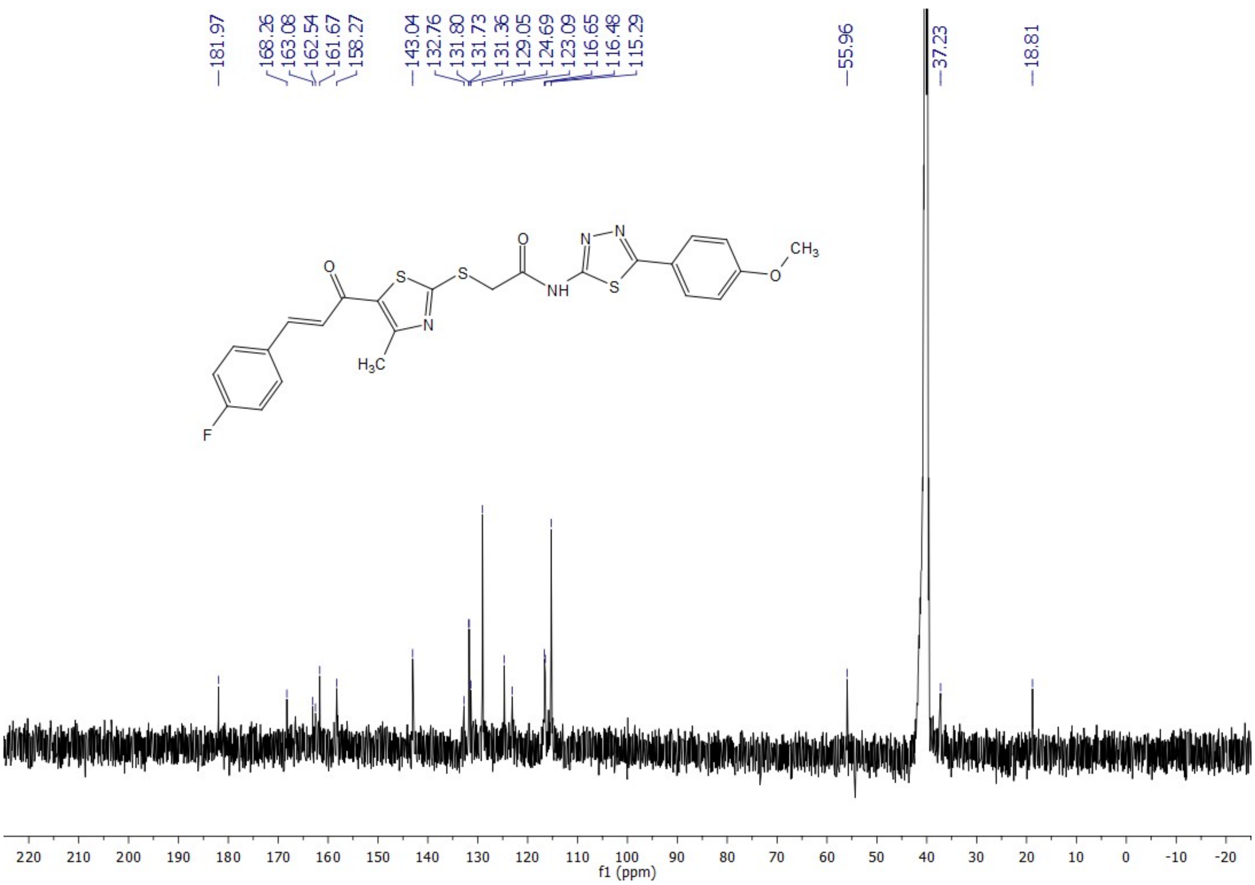

Figure S25. ^{13}C NMR spectrum of compound **9m**

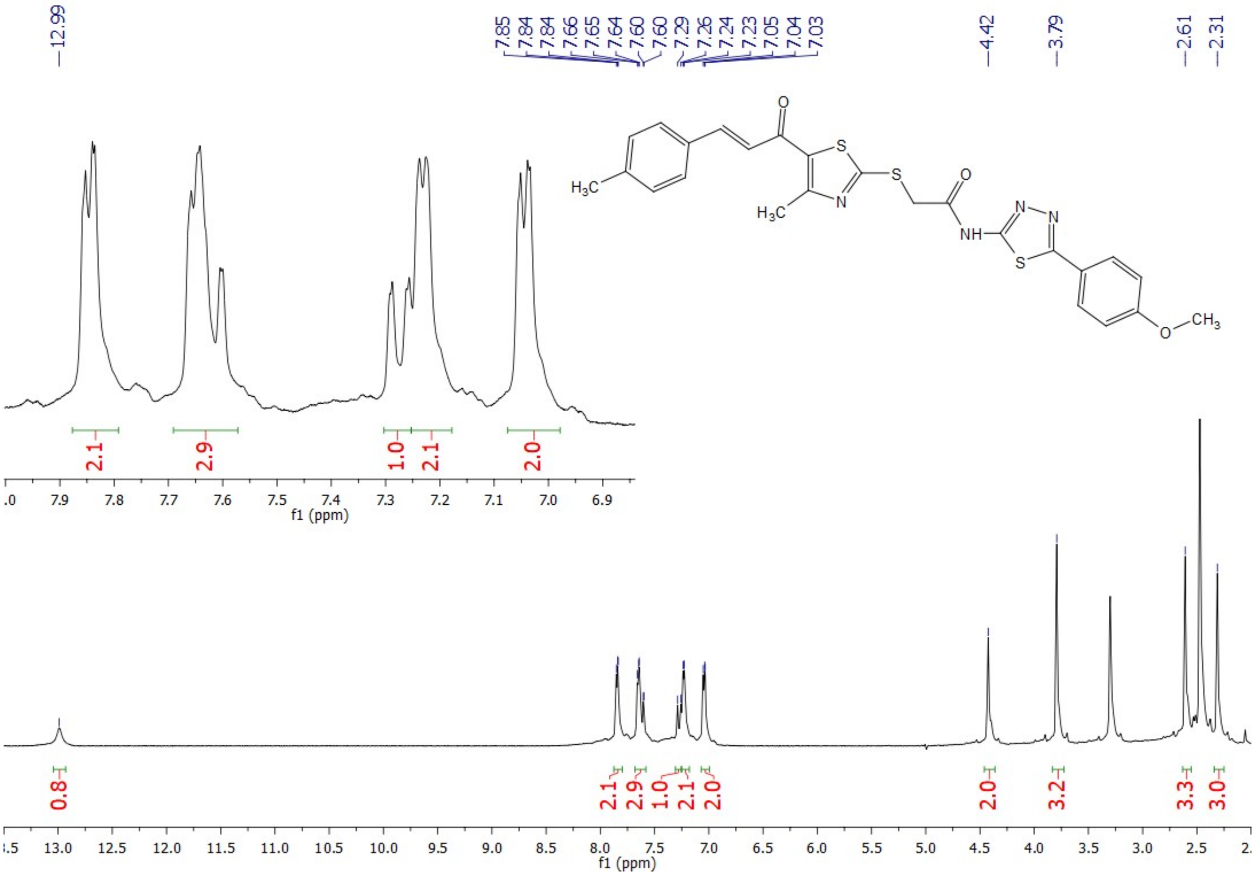

Figure S26. ^{1}H NMR spectrum of compound **9n**

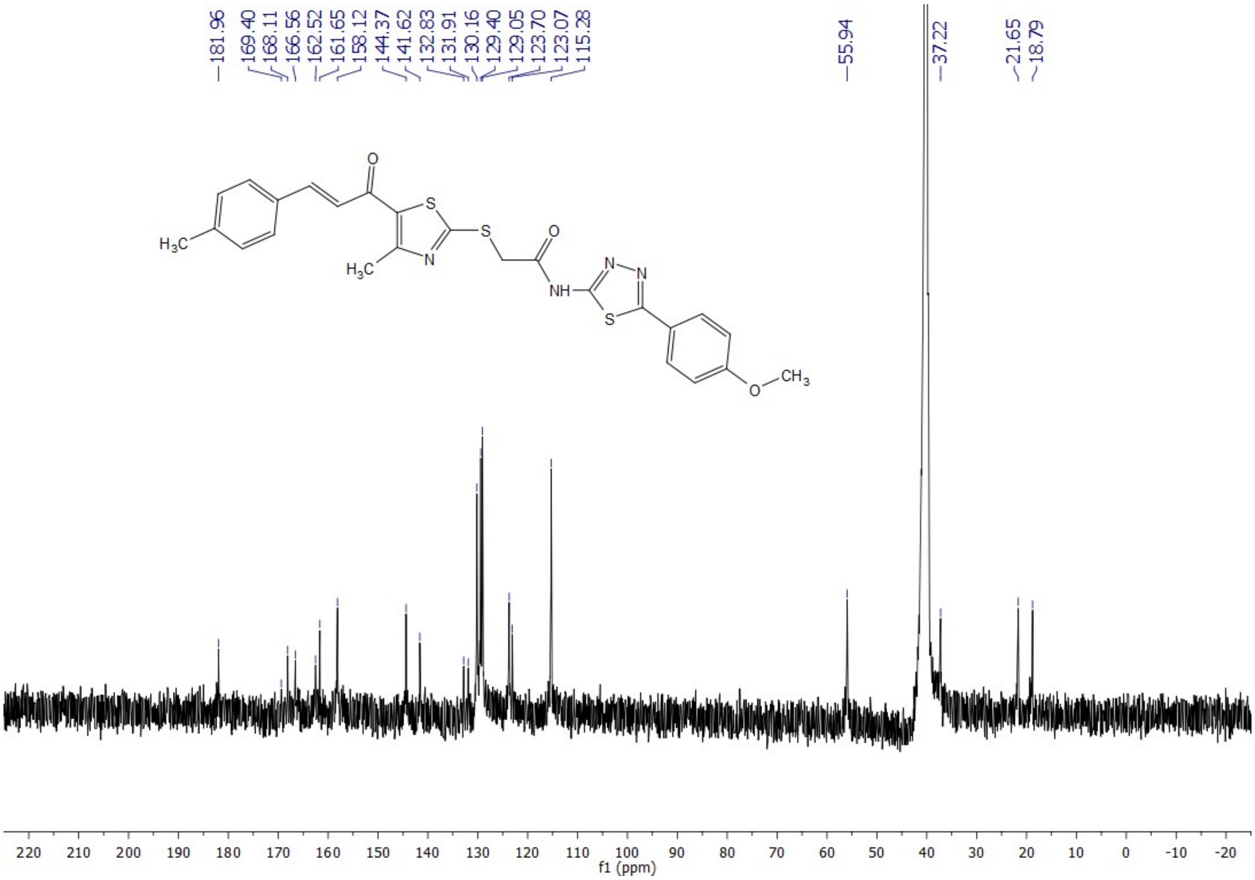

Figure S27. ^{13}C NMR spectrum of compound **9n**

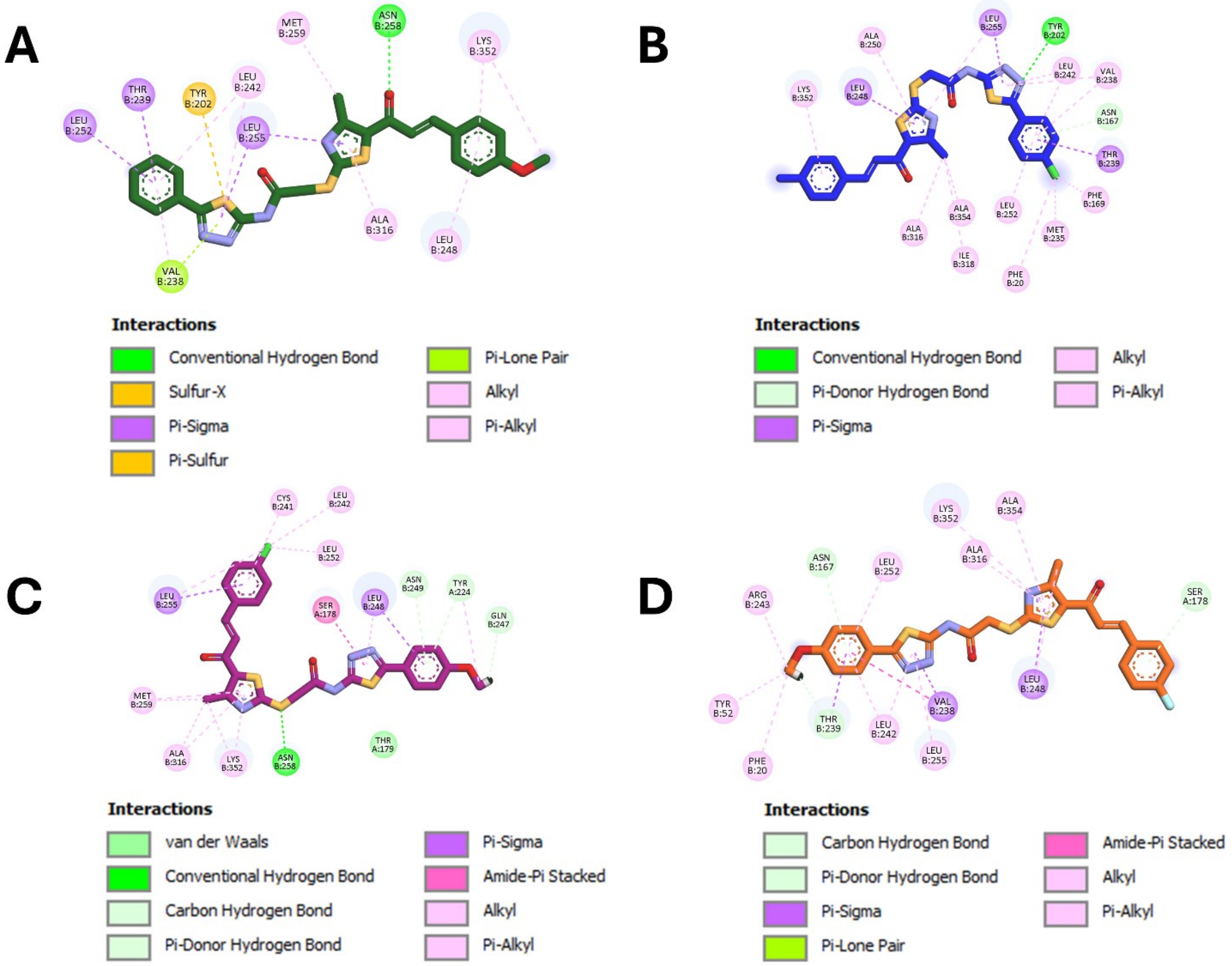

Figure S28. 2D interaction diagrams of (A) compound **9e**, (B) compound **9i**, (C) compound **9l**, and (D) compound **9m** within the colchicine binding site of tubulin.

Table S1. Docking interactions of compound **9k**

Compound	Binding Affinity (kcal/mol)	Non-Classical Hydrogen Bonding	Hydrophobic Interactions
**9k**	-9.1	Thr239, Gln136	Thr239, Leu242, Leu248, Leu252, Leu255, Ala316, Ala354, Cys241, Ile318, Ile4, Ala250, Lys352, Val238

Table S2. Physicochemical Properties of compound **9k**

Property	9k	Comment
Molecular Weight	508.07	Contain hydrogen atoms. Optimal:100~600
Volume	481.045	Van der Waals volume
Density	1.056	Density = MW / Volume
nHA	7.0	Number of hydrogen bond acceptors. Optimal:0~12
nHD	1.0	Number of hydrogen bond donors. Optimal:0~7
nRot	10.0	Number of rotatable bonds. Optimal:0~11
nRing	4.0	Number of rings. Optimal:0~6
MaxRing	6.0	Number of atoms in the biggest ring. Optimal:0~18
nHet	10.0	Number of heteroatoms. Optimal:1~15
fChar	0.0	Formal charge. Optimal:-4 ~4
nRig	25.0	Number of rigid bonds. Optimal:0~30
Flexibility	0.4	Flexibility = nRot /nRig
Stereo Centers	0.0	Stereo Centers. Optimal: □ 2
TPSA	94.07	Topological Polar Surface Area. Optimal:0~140
logS	-5.84	The logarithm of aqueous solubility value.
logP	3.924	The logarithm of the n-octanol/water distribution coefficients at pH=7.4.
logD	3.721	The logarithm of the n-octanol/water distribution coefficient.
pka (Acid)	8.632	Acid-base dissociation constant (pKa) value represents the strength of a drug molecule's acidity or basicity.
pka (Base)	2.248	Acid-base dissociation constant (pKa) value represents the strength of a drug molecule's acidity or basicity.
Melting point	155.493	The predicted melting point of a compound is expressed in degrees Celsius (°C).<br>Melting points below 25°C are classified as liquids, while melting points above 25°C are classified as solids.
Boiling point	363.153	The predicted melting point of a compound is expressed in degrees Celsius (°C).<br>A normal boiling point below 25°C is categorized as a gas.

Table S3. Medicinal Chemistry of compound **9k**

**Property**	**9l**	**Comment**
QED	0.178	■ A measure of drug-likeness based on the concept of desirability;<br>■ Attractive: > 0.67;<br>■ unattractive: 0.49~0.67;<br>■ too complex: < 0.34
GASA	0.0	■ ES: Easy to synthesize; HS: Hard to synthesize;<br>■ The output value represents the probability of being difficult to synthesize, ranging from 0 to 1.
Synth	2.0	■ Synthetic accessibility score is designed to estimate ease of synthesis of drug-like molecules.<br>■ SAscore □ 6, difficult to synthesize; SAscore <6, easy to synthesize
Fsp3	0.125	■ The number of sp3 hybridized carbons / total carbon count, correlating with melting point and solubility.<br>■ Fsp^3 □0.42 is considered a suitable value.
MCE-18	22.0	■ MCE-18 stands for medicinal chemistry evolution.<br>■ MCE-18□45 is considered a suitable value.
NPscore	-1.794	■ Natural product-likeness score.<br>■ This score is typically in the range from -5 to 5.<br>■ The higher the score is, the higher the probability is that the molecule is a NP.
Lipinski Rule	0.0	■ MW □ 500; logP □ 5; Hacc □ 10; Hdon □ 5<br>■ If two properties are out of range, a poor absorption or permeability is possible, one is acceptable.
Pfizer Rule	0.0	■ logP > 3; TPSA < 75<br>■ Compounds with a high log P (>3) and low TPSA (<75) are likely to be toxic.
GSK Rule	1.0	■ MW □ 400; logP □ 4<br>■ Compounds satisfying the GSK rule may have a more favorable ADMET profile
Golden Triangle	1.0	■ 200 □ MW □ 500; -2 □ logD □ 5<br>■ Compounds satisfying the Golden Triangle rule may have a more favorable ADMET profile.

PAINS	0 alerts	frequent hitters, Alpha-screen artifacts and reactive compound 480 substructures (J Med Chem 201053:2719-40)
ALARM NMR	6 alerts	Thiol reactive compounds.
BMS	0 alerts	undesirable, reactive compounds 176 substructures (J Chem Inf Model 200646:1060-8)
Chelator Rule	0 alerts	Chelating compounds.
Colloidal aggregators	1.0	■ Category 0: non-colloidal aggregators; ■ Category 1: colloidal aggregators. ■ The output value is the probability of being colloidal aggregators, within the range of 0 to 1.
FLuc inhibitors	0.997	■ Category 0: non-fLuc inhibitors; ■ Category 1: fLuc inhibitors. The output value is the probability of being fLuc inhibitors, within the range of 0 to 1.
Blue fluorescence	0.952	■ Category 0: non-blue fluorescence; ■ Category 1: blue fluorescence. The output value is the probability of being blue fluorescence, within the range of 0 to 1.
Green fluorescence	1.0	■ Category 0: non-green fluorescence; ■ Category 1: green fluorescence. The output value is the probability of being green fluorescence, within the range of 0 to 1.
Reactive compounds	0.026	■ Category 0: non-reactive compound; ■ Category 1: reactive compound. The output value is the probability of being reactive compound, within the range of 0 to 1.
Promiscuous compounds	0.0	■ Category 0: non-promiscuous compound; ■ Category 1: promiscuous compound. The output value is the probability of being promiscuous compound, within the range of 0 to 1.

Table S4. Absorption of compound **9k**

**Property**	**9k**	**Comment**
Caco-2 Permeability	-5.269	Optimal: higher than -5.15 Log unit
MDCK Permeability	-4.674	■ low permeability: < 2 × 10-6 cm/s<br>■ medium permeability: 2-20 × 10^{-6} cm/s<br>■ high passive permeability: > 20 × 10^{-6} cm/s
PAMPA	0.06	■ The experimental data for Peff was logarithmically transformed (logPeff).<br>■ Molecules with log Peff values below 2.0 were classified as low-permeability (Category 0), while those with log Peff values exceeding 2.5 were classified as high-permeability (Category 1).
Pgp-inhibitor	0.844	■ Category 1: Inhibitor;<br>■ Category 0: Non-inhibitor;<br>■ The output value is the probability of being Pgp-inhibitor
Pgp-substrate	0.0	■ Category 1: substrate;<br>■ Category 0: Non-substrate;<br>■ The output value is the probability of being Pgp-substrate
HIA	0.011	■ Human Intestinal Absorption<br>■ Category 1: HIA+(HIA < 30%);<br>■ Category 0: HIA-(HIA >= 30%);<br>■ The output value is the probability of being HIA+
$F_{20\%}$	0.011	■ 20% Bioavailability<br>■ Category 1: F 20% + (bioavailability < 20%);<br>■ Category 0: F 20% - (bioavailability 20%);<br>■ The output value is the probability of being F 20%<br>+
$F_{30\%}$	0.677	■ 30% Bioavailability<br>■ Category 1: F 30% + (bioavailability < 30%);<br>■ Category 0: F 30% - (bioavailability 30%);<br>■ The output value is the probability of being F 30%<br>+
$F_{50\%}$	0.264	■ 50% Bioavailability<br>■ Category 1: F 50% + (bioavailability < 50%);<br>■ Category 0: F 50% - (bioavailability 50%);<br>■ The output value is the probability of being F 50%<br>+

Table S5. Distribution of compound **9k**

**Property**	**9k**	**Comment**
PPB	98.768	■ Plasma Protein Binding Optimal: < 90%.<br>■ Drugs with high protein-bound may have a low therapeutic index.
VDss	-0.011	■ Volume Distribution<br>■ Optimal: 0.04-20L/kg
BBB	0.007	■ Blood-Brain Barrier Penetration<br>■ Category 1: BBB+; Category 0: BBB-;<br>■ The output value is the probability of being BBB+
Fu	1.082	■ The fraction unbound in plasms<br>■ Low: <5%; Middle: 5~20%; High: > 20%
OATP1B1 inhibitor	1.0	■ Category 0: Non-inhibitor; Category 1: inhibitor.<br>■ The output value is the probability of being inhibitor, within the range of 0 to 1.
OATP1B3 inhibitor	0.995	■ Category 0: Non-inhibitor; Category 1: inhibitor.<br>■ The output value is the probability of being inhibitor, within the range of 0 to 1.
BCRP inhibitor	0.0	■ Category 0: Non-inhibitor; Category 1: inhibitor.<br>■ The output value is the probability of being inhibitor, within the range of 0 to 1.
MRP1 inhibitor	0.869	■ Category 0: Non-inhibitor; Category 1: inhibitor.<br>■ The output value is the probability of being inhibitor, within the range of 0 to 1.

Table S6. Metabolism of compound **9k**

Property	9k	Comment
CYP1A2 inhibitor	1.0	■ Category 1: Inhibitor; Category 0: Non-inhibitor; ■ The output value is the probability of being inhibitor.
CYP1A2 substrate	0.0	■ Category 1: Substrate; Category 0: Non-substrate; The output value is the probability of being substrate.
CYP2C19 inhibitor	1.0	■ Category 1: Inhibitor; Category 0: Non-inhibitor; The output value is the probability of being inhibitor.
CYP2C19 substrate	0.0	■ Category 1: Substrate; Category 0: Non-substrate; The output value is the probability of being substrate.
CYP2C9 inhibitor	1.0	■ Category 1: Inhibitor; Category 0: Non-inhibitor; The output value is the probability of being inhibitor.
CYP2C9 substrate	0.988	■ Category 1: Substrate; Category 0: Non-substrate; The output value is the probability of being substrate.
CYP2D6 inhibitor	0.167	■ Category 1: Inhibitor; Category 0: Non-inhibitor; The output value is the probability of being inhibitor.
CYP2D6 substrate	0.228	■ Category 1: Substrate; Category 0: Non-substrate; The output value is the probability of being substrate.
CYP3A4 inhibitor	0.984	■ Category 1: Inhibitor; Category 0: Non-inhibitor; The output value is the probability of being inhibitor.
CYP3A4 substrate	0.003	■ Category 1: Substrate; Category 0: Non-substrate; The output value is the probability of being substrate.
CYP2B6 inhibitor	1.0	■ Category 1: Inhibitor; Category 0: Non-inhibitor; The output value is the probability of being

		inhibitor.
CYP2B6 substrate	0.0	■ Category 1: Substrate; Category 0: Non-substrate;<br>The output value is the probability of being substrate.
CYP2C8 inhibitor	1.0	■ Category 1: Inhibitor; Category 0: Non-inhibitor;<br>The output value is the probability of being inhibitor.
HLM Stability	0.841	■ human liver microsomal (HLM) stability<br>Category 0: stable+ (HLM > 30 min); Category 1: unstable- (HLM □ 30 min). The output value is the probability of human liver microsomal instability, where a value closer to 1 indicates a higher likelihood of instability. The range is between 0 and 1.

Table S7. Excretion of compound **9k**

Property	9k	Comment
CL_{plasma}	2.29	■ The unit of predicted CLplasma penetration is ml/min/kg. >15 ml/min/kg: high clearance; 5-15 ml/min/kg: moderate clearance; < 5 ml/min/kg: low clearance.
$T_{1/2}$	1.156	■ The unit of predicted T1/2 is hours.<br>■ ultra-short half-life drugs: 1/2 < 1 hour; short half-life drugs: T1/2 between 1-4 hours; intermediate short half-life drugs: T1/2 between 4-8 hours; long half-life drugs: T1/2 > 8 hours.

Table S8. Toxicity of compound **9k**

Property	9k	Comment
hERG Blockers	0.598	■ Molecules with IC50 □10□M or □50% inhibition at 10 □M were classified as hERG+ (Category 1),<br>■ while molecules with IC50 >10□M or < 50% inhibition at 10□M were classified as hERG - (Category 0).<br>■ The output value is the probability of being hERG+, within the range of 0 to 1.
hERG Blockers (10um)	0.477	■ Molecules with IC50 □10 □M are classified as hERG+ (Category 1),<br>■ and molecules with IC50 > 10□M are classified as hERG- (Category 0).

		■ The output value is the probability of being hERG+, within the range of 0 to 1.
DILI	0.999	■ Drug Induced Liver Injury.<br>■ Category 1: drugs with a high risk of DILI;<br>■ Category 0: drugs with no risk of DILI.<br>■ The output value is the probability of being toxic.
AMES Mutagenicity	0.537	■ AMES Toxicity<br>■ Category 1: Ames positive(+);<br>■ Category 0: Ames negative(-);<br>■ The output value is the probability of being toxic.
Rat Oral Acute Toxicity	0.185	■ Rat Oral Acute Toxicity.<br>■ Category 0: low-toxicity, > 500 mg/kg;<br>■ Category 1: high-toxicity; < 500 mg/kg.<br>■ The output value is the probability of being toxic, within the range of 0 to 1.
FDAMDD	0.11	■ FDA Maximum (Recommended) Daily Dose.<br>■ Category 1: FDAMDD (+);<br>■ Category 0: FDAMDD (-);<br>The output value is the probability of being positive.
Skin Sensitization	0.912	■ Category 1: Sensitizer;<br>■ Category 0: Non-sensitizer.<br>■ The output value is the probability of being toxic, within the range of 0 to 1.
Carcinogenicity	0.815	■ Category 1: carcinogens;<br>■ Category 0: non-carcinogens;<br>■ The output value is the probability of being toxic.
Eye Corrosion	0.0	■ Eye Corrosion<br>■ Category 1: corrosives; Category 0: noncorrosives;<br>The output value is the probability of being corrosives.
Eye Irritation	0.085	■ Eye Irritation<br>■ Category 1: irritants; Category 0: nonirritants;<br>The output value is the probability of being irritants.
Respiratory	0.198	■ Category 1: respiratory toxicants;<br>■ Category 0: non-respiratory toxicants.<br>The output value is the probability of being toxic, within the range of 0 to 1.
Human Hepatotoxicity	0.77	■ Human Hepatotoxicity<br>■ Category 1: H-HT positive(+);

		■ Category 0: H-HT negative(-);<br>The output value is the probability of being toxic.
Drug-induced Nephrotoxicity	0.852	■ Category 0: non-nephrotoxic (-);<br>■ Category 1: nephrotoxic (+).<br>The output value is the probability of being nephrotoxic (+), within the range of 0 to 1.
Ototoxicity	0.496	■ Category 0: non-ototoxicity (-);<br>■ Category 1: ototoxicity (+).<br>The output value is the probability of being ototoxicity (+), within the range of 0 to 1.
Hematotoxicity	0.143	■ Category 0: non-hematotoxicity (-);<br>■ Category 1: hematotoxicity (+).<br>The output value is the probability of being hematotoxicity (+), within the range of 0 to 1.
Genotoxicity	0.999	■ Category 0: non-Genotoxicity (-);<br>■ Category 1: Genotoxicity (+).<br>The output value is the probability of being ototoxicity (+), within the range of 0 to 1.
RPMI-8226 Immunitoxicity	0.088	■ Category 0: non-cytotoxicity (-);<br>■ Category 1: cytotoxicity (+).<br>The output value is the probability of being ototoxicity (+), within the range of 0 to 1.
A549 Cytotoxicity	0.127	■ Category 0: non-cytotoxicity (-);<br>■ Category 1: cytotoxicity (+).<br>The output value is the probability of being ototoxicity (+), within the range of 0 to 1.
Hek293 Cytotoxicity	0.691	■ Category 0: non-cytotoxicity (-);<br>■ Category 1: cytotoxicity (+).<br>The output value is the probability of being ototoxicity (+), within the range of 0 to 1.
Drug-induce d Neurotoxi city	0.609	■ Category 0: non-neurotoxic (-);<br>■ Category 1: neurotoxic (+).<br>The output value is the probability of being neurotoxic (+), within the range of 0 to 1.

Table S9. Environmental toxicity of compound **9k**

**Property**	**9k**	**Comment**
Bioconcentraion Factors	1.198	■ Bioconcentration factors are used for considering secondary poisoning potential and assessing risks to human health via the food chain.<br>The unit is □log10[(mg/L)/(1000*MW)]
IGC_{50}	4.786	■ Tetrahymena pyriformis 50 percent growth inhibition concentration.<br>The unit is □log10[(mg/L)/(1000*MW)]
$LC_{50}FM$	6.285	■ 96-hour fathead minnow 50 percent lethal concentration.<br>The unit is □log10[(mg/L)/(1000*MW)]
$LC_{50}DM$	6.408	■ 48-hour daphnia magna 50 percent lethal concentration.<br>The unit is □log10[(mg/L)/(1000*MW)]

Table S10. Tox21 pathway of compound **9k**

**Property**	**9k**	**Comment**
NR-AhR	0.509	■ Aryl hydrocarbon receptor<br>■ Category 1: actives ;<br>■ Category 0: inactives;<br>■ The output value is the probability of being active.
NR-AR	0.0	■ Androgen receptor<br>■ Category 1: actives ;<br>■ Category 0: inactives;<br>■ The output value is the probability of being active.
NR-AR-LBD	0.032	■ Androgen receptor ligand-binding domain<br>■ Category 1: actives ;<br>■ Category 0: inactives;<br>■ The output value is the probability of being active.
NR-Aromatase	0.028	■ Category 1: actives ;<br>■ Category 0: inactives;<br>■ The output value is the probability of being active.
NR-ER	0.514	■ Estrogen receptor<br>■ Category 1: actives ;<br>■ Category 0: inactives;<br>■ The output value is the probability of being active.
NR-ER-LBD	0.0	■ Estrogen receptor ligand-binding domain

		■ Category 1: actives ;<br>■ Category 0: inactives;<br>■ The output value is the probability of being active.
NR-PPAR-gamma	0.345	■ Peroxisome proliferator-activated receptor gamma<br>■ Category 1: actives ;<br>■ Category 0: inactives;<br>■ The output value is the probability of being active.
SR-ARE	0.997	■ Antioxidant response element<br>■ Category 1: actives ;<br>■ Category 0: inactives;<br>■ The output value is the probability of being active.
SR-ATAD5	0.065	■ ATPase family AAA domain-containing protein 5<br>■ Category 1: actives ;<br>■ Category 0: inactives;<br>The output value is the probability of being active.
SR-HSE	0.294	■ Heat shock factor response element<br>■ Category 1: actives ;<br>■ Category 0: inactives;<br>The output value is the probability of being active.
SR-MMP	0.986	■ Mitochondrial membrane potential<br>■ Category 1: actives ;<br>■ Category 0: inactives;<br>The output value is the probability of being active.
SR-p53	0.819	■ p53, a tumor suppressor protein<br>■ Category 1: actives ;<br>■ Category 0: inactives;<br>The output value is the probability of being active.

Table S11. Toxicophore Rules of compound **9k**

**Property**	**9k**	**Comment**
Acute Toxicity Rule	0	■ 20 substructures;<br>acute toxicity during oral administration
Genotoxic Carcinogenicity Rule	2 alerts	■ 117 substructures;<br>carcinogenicity or mutagenicity
NonGenotoxic Carcinogenicity Rule	1 alerts	■ 23 substructures;<br>carcinogenicity through nongenotoxic mechanisms
Skin Sensitization Rule	6 alerts	■ 155 substructures;<br>skin irritation
Aquatic Toxicity Rule	2 alerts	■ 99 substructures;<br>toxicity to liquid(water)
NonBiodegradale Rule	0	■ 19 substructures;<br>non-biodegradable
SureChEMBL Rule	0	■ 164 substructures;<br>MedChem unfriendly status
FAF-Drugs4 Rule	3 alerts	154 toxic substructures from FAF-Drug4

4.2. Biological evaluation

4.2.1 Cell Viability assay (MTT assay)

MTT assay was performed to investigate the effect of the synthesized compounds on mammary epithelial cells (MCF-10A). The cells were propagated in medium consisting of Ham's F-12 medium/ Dulbecco's modified Eagle's medium (DMEM) (1:1) supplemented with 10% foetal calf serum, 2 mM glutamine, insulin (10 μg/mL), hydrocortisone (500 ng/mL) and epidermal growth factor (20 ng/mL). Trypsin ethylenediamine tetra acetic acid (EDTA) was used to passage the cells after every 2-3 days. 96-well flat-bottomed cell culture plates were used to seed the cells at a density of 104 cells mL^{-1}. The medium was aspirated from all the wells of culture plates after 24 h followed by the addition of synthesized compounds (in 200 μL medium to yield a final concentration of 0.1% (v/v) dimethyl sulfoxide) into individual wells of the plates. Four wells were designated to a single compound. The plates were allowed to incubate at 37°C for 96 h. Afterwards, the medium was aspirated and 3-[4,5-dimethylthiazol-2-yl]-2,5-diphenyltetrazolium bromide (MTT) (0.4 mg/mL) in medium was added to each well and subsequently incubated for 3 h. The medium was aspirated and 150 μL dimethyl sulfoxide (DMSO) was added to each well. The plates were vortexed followed by the measurement of absorbance at 540 nm on a microplate reader. The results were presented as inhibition (%) of proliferation in contrast to controls comprising 0.1% DMSO.

4.2.2. Assay for antiproliferative effect

To explore the antiproliferative potential of compounds propidium iodide fluorescence assay was performed using different cell lines such as A549 (Human Lung Adenocarcinoma), HeLa (Human Cervical Cancer), and HCT 116 (Human Colorectal Adenocarcinoma), respectively. To calculate the total nuclear DNA, a fluorescent dye (propidium iodide, PI) is used which can attach to the

DNA, thus offering a quick and precise technique. PI cannot pass through the cell membrane and its signal intensity can be considered as directly proportional to quantity of cellular DNA. Cells whose cell membranes are damaged or have changed permeability are counted as dead ones. The assay was performed by seeding the cells of different cell lines at a density of 3000-7500 cells/well (in200µl medium) in culture plates followed by incubation for 24h at 37 °C in humidified 5%CO2/95% air atmospheric conditions. The medium was removed; the compounds were added to the plates at 10 µM concentrations (in 0.1% DMSO) in triplicates, followed by incubation for 48h. DMSO (0.1%) was used as control. After incubation, medium was removed followed by the addition of PI (25 µl, 50µg/mL in water/medium) to each well of the plates. At -80 °C, the plates were allowed to freeze for 24 h, followed by thawing at 25 ^{o}C. A fluorometer (Polar-Star BMG Tech) was used to record the readings at excitation and emission wavelengths of 530 and 620 nm for each well. The percentage cytotoxicity of compounds was calculated using the following formula:

$$\% \text{ Cytotoxicity} = \frac{A_C - A_{TC}}{A_C} \times 100$$

Where A*TC*= Absorbance of treated cells and AC= Absorbance of control. Erlotinib was used as positive control in the assay.

4.2.3. Tubulin polymerization assay

The activity of compounds on tubulin polymerization was investigated by Tubulin Polymerization Assay Kit (Cytoskeleton Inc., Denver, CO, USA), which works via fluorescent reporter enhancement. The fluorescence of compounds (dissolved in DMSO at 5 and 25 µM concentration) was recorded in triplicates using FLUO star OPTIMA. Docetaxel and vincristine (Apoteket AB,

Sweden) served as positive stabilizing and destabilizing controls. Both were used at 3 µM concentration in PBS.

4.2.4. Caspase-3 activation assay

Allow all reagents to reach room temperature before use. Gently mix all liquid reagents prior to use. Determine the number of 8-well strips needed for the assay. Insert these in the frame(s) for current use. Add 100 µl of the *Standard Diluent Buffer* to the zero standard wells. Well(s) reserved for chromogen blank should be left empty. Add 100 µl of standards and controls or diluted samples to the appropriate microtiter wells. The sample dilution chosen should be optimized for each experimental system. Tap gently on side of plate to mix. Cover wells with *plate cover* and incubate for 2 hours at room temperature. Thoroughly aspirate or decant solution from wells and discard the liquid, Wash wells 4 times. Pipette 100 µl of *Caspase-3 (Active) Detection Antibod*y solution into each well except the chromogen blank(s). Tap gently on the side of the plate to mix. Cover plate with *plate cover* and incubate for 1 hour at room temperature. Thoroughly aspirate or decant solution from wells and discard the liquid, Wash wells 4 times. Add 100 µl Anti-Rabbit IgG HRP Working Solution to each well except the chromogen blank(s). Prepare the working dilution as described in Preparing IgG HRP. Cover wells with the *plate cover* and incubate for 30 minutes at room temperature. Thoroughly aspirate or decant solution from wells and discard the liquid. Wash wells 4 times. Add 100 µl of *Stabilized Chromogen* to each well. The liquid in the wells will begin to turn blue. Incubate for 30 minutes at room temperature and in the dark. The incubation time for chromogen substrate is often determined by the microtiter plate reader used. Many plate readers have the capacity to record a maximum optical density (O.D.) of 2.0. The O.D. values should be monitored, and the substrate reaction stopped before the O.D. of the positive wells exceeds the limits of the instrument. The O.D. values at 450 nm can only be read after the *Stop Solution* has

been added to each well. If using a reader that records only to 2.0 O.D., stopping the assay after 20 to 25 minutes is suggested. Add 100 µl of *Stop Solution* to each well. Tap side of plate gently to mix. The solution in the wells should change from blue to yellow. Read the absorbance of each well at 450 nm having blanked the plate reader against a chromogen blank composed of 100 µl each of *Stabilized Chromogen* and *Stop Solution*. Read the plate within 2 hours after adding the *Stop Solution*. Use a curve fitting software to generate the standard curve. A four-parameter algorithm provides the best standard curve fit. Read the concentrations for unknown samples and controls from the standard curve. Multiply value(s) obtained for sample(s) by the appropriate dilution factor to correct for the dilution in step 3. Samples producing signals greater than that of the highest standard should be diluted in *Standard Diluent Buffer* and reanalyzed.

4.2.5. Caspase-8/9 activation assay

Cells were obtained from American Type Culture Collection, cells were grown in RPMI 1640 containing 10% fetal bovine serum at 37°C, stimulated with the compounds to be tested for caspase 8/9, and lysed with Cell Extraction Buffer. This lysate was diluted in Standard Diluent Buffer over the range of the assay and measured for human active caspase-8/9 content. (*Cells are Plated in a density of 1.2 – 1.8 × 10,000 cells/well in a volume of 100µl complete growth medium + 100 ul of the tested compound per well in a 96-well plate for 24 hours before the enzyme assay*). The absorbance of each microwell was read on a spectro-photometer at 450 nm. A standard curve is prepared from 7 human Caspase-8/9 standard dilutions and human Caspase-8/9 concentration determined.

4.2.6. Bax activation assay

Bring all reagents, except the human Bax-α Standard, to room temperature for at least 30 minutes prior to opening. The human Bax-α Standard solution should not be left at room temperature for more than 10 minutes. All standards, controls and samples should be run in duplicate. Refer to the Assay Layout Sheet to determine the number of wells to be used and put any remaining wells with the desiccant back into the pouch and seal the ziploc. Store unused wells at 4 °C. Pipet 100 µL of Assay Buffer into the S0 (0 pg/mL standard) wells. Pipet 100 µL of Standards #1 through #6 into the appropriate wells. Pipet 100 µL of the Samples into the appropriate wells. Tap the plate gently to mix the contents. Seal the plate and incubate at room temperature on a plate shaker for 1 hour at ~500 rpm. Empty the contents of the wells and wash by adding 400 µL of wash solution to every well. Repeat the wash 4 more times for a total of **5 washes**. After the final wash, empty or aspirate the wells and firmly tap the plate on a lint free paper towel to remove any remaining wash buffer. Pipet 100 µL of yellow Antibody into each well, except the Blank. Seal the plate and incubate at room temperature on a plate shaker for 1 hour at ~500 rpm. Empty the contents of the wells and wash by adding 400 µL of wash solution to every well. Repeat the wash 4 more times for a total of **5** washes. After the final wash, empty or aspirate the wells and firmly tap the plate on a lint free paper towel to remove any remaining wash buffer. Add 100 µL of blue Conjugate to each well, except the Blank. Seal the plate and incubate at room temperature on a plate shaker for 30 minutes at ~500 rpm. Empty the contents of the wells and wash by adding 400 µL of wash solution to every well. Repeat the wash 4 more times for a total of **5 washes**. After the final wash, empty or aspirate the wells and firmly tap the plate on a lint free paper towel to remove any remaining wash buffer. Pipet 100 µL of Substrate Solution into each well. Incubate for 30 minutes at room temperature on a plate shaker at ~500 rpm. Pipet 100 µL Stop Solution to each well. Blank the plate reader against

the Blank wells, read the optical density at 450 nm. Calculate the average net Optical Density (OD) bound for each standard and sample by subtracting the average Blank OD from the average OD for each standard and sample. Using linear graph paper, plot the Average Net OD for each standard versus Bax concentration in each standard. Approximate a straight line through the points. The concentration of Bax in the unknowns can be determined by interpolation.

4.2.7. Bcl-2 inhibition assay

Mix all the reagents thoroughly without foaming before use. Wash the microwells twice with approximately 300 µL Wash Buffer per well with thorough aspiration of microwell contents between washes. Take caution not to scratch the surface of the microwells. After the last wash, empty the wells and tap microwell strips on absorbent pad or paper towel to remove excess Wash Buffer. Use the microwell strips immediately after washing or place upside down on a wet absorbent paper for not longer than 15 minutes. Do not allow wells to dry. Add 100 µL of Sample Diluent in duplicate to all standard wells and to the blank wells. Prepare standard (1:2 dilution) in duplicate ranging from 32 ng/mL to 0.5 ng/mL. Add 100 µL of Sample Diluent, in duplicate, to the blank wells. Add 80 µL of Sample Diluent, in duplicate, to the sample wells. Add 20 µL of each Sample, in duplicate, to the designated wells. Add 50 µL of diluted biotin-conjugate to all wells, including the blank wells. Cover with a plate cover and incubate at room temperature, on a microplate shaker at 100 rpm if available, for 2 hours. Remove plate cover and empty the wells. Wash microwell strips 3 times as described in step 2. Add 100 µL of diluted Streptavidin-HRP to all wells, including the blank wells. Cover with a plate cover and incubate at room temperature, on a microplate shaker at 100 rpm if available, for 1 hour. Remove the plate cover and empty the wells. Wash microwell strips 3 times as described in step 2. Proceed to the next step. Pipette 100 µl of mixed TMB Substrate Solution to all wells, including the blanks. Incubate the microwell

strips at room temperature (18° to 25°C) for about 15 minutes, if available on a rotator set at 100 rpm. Avoid direct exposure to intense light. The point, at which the substrate reaction is stopped, is often determined by the ELISA reader. Many ELISA readers record absorbance only up to 2.0 O.D. Therefore, the color development within individual microwells must be watched by the person running the assay and the substrate reaction stopped before positive wells are no longer properly detectable. Stop the enzyme reaction by quickly pipetting 100 µL of Stop Solution into each well, including the blank wells. It is important that the Stop Solution is spread quickly and uniformly throughout the microwells to completely inactivate the enzyme. Results must be read immediately after the Stop Solution is added or within one hour if the microwell strips are stored at 2 - 8°C in the dark. Read the absorbance of each microwell on a spectrophotometer using 450 nm as the primary wavelength.
